# Imaging suicidal thoughts and behaviors: a comprehensive review of 2 decades of neuroimaging studies

**DOI:** 10.1038/s41380-019-0587-x

**Published:** 2019-12-02

**Authors:** Lianne Schmaal, Anne-Laura van Harmelen, Vasiliki Chatzi, Elizabeth T. C. Lippard, Yara J. Toenders, Lynnette A. Averill, Carolyn M. Mazure, Hilary P. Blumberg

**Affiliations:** 1 Orygen, The National Centre of Excellence in Youth Mental Health, Parkville, VIC Australia; 2 Centre for Youth Mental Health, The University of Melbourne, Parkville, VIC Australia; 3 Department of Psychiatry, University of Cambridge, Cambridge, UK; 4 Psychiatry, Dell Medical School, University of Texas, Austin, TX USA; 5 Psychiatry, Yale School of Medicine, New Haven, CT USA; 6 Department of Veterans Affairs National Center for PTSD, Clinical Neurosciences Division, West Haven, CT USA; 7 Psychiatry and Women’s Health Research at Yale, Yale School of Medicine, New Haven, CT USA; 8 Psychiatry, Radiology and Biomedical Imaging, Child Study Center, Yale School of Medicine, New Haven, CT USA

**Keywords:** Neuroscience, Predictive markers

## Abstract

Identifying brain alterations that contribute to suicidal thoughts and behaviors (STBs) are important to develop more targeted and effective strategies to prevent suicide. In the last decade, and especially in the last 5 years, there has been exponential growth in the number of neuroimaging studies reporting structural and functional brain circuitry correlates of STBs. Within this narrative review, we conducted a comprehensive review of neuroimaging studies of STBs published to date and summarize the progress achieved on elucidating neurobiological substrates of STBs, with a focus on converging findings across studies. We review neuroimaging evidence across differing mental disorders for structural, functional, and molecular alterations in association with STBs, which converges particularly in regions of brain systems that subserve emotion and impulse regulation including the ventral prefrontal cortex (VPFC) and dorsal PFC (DPFC), insula and their mesial temporal, striatal and posterior connection sites, as well as in the connections between these brain areas. The reviewed literature suggests that impairments in medial and lateral VPFC regions and their connections may be important in the excessive negative and blunted positive internal states that can stimulate suicidal ideation, and that impairments in a DPFC and inferior frontal gyrus (IFG) system may be important in suicide attempt behaviors. A combination of VPFC and DPFC system disturbances may lead to very high risk circumstances in which suicidal ideation is converted to lethal actions via decreased top-down inhibition of behavior and/or maladaptive, inflexible decision-making and planning. The dorsal anterior cingulate cortex and insula may play important roles in switching between these VPFC and DPFC systems, which may contribute to the transition from suicide thoughts to behaviors. Future neuroimaging research of larger sample sizes, including global efforts, longitudinal designs, and careful consideration of developmental stages, and sex and gender, will facilitate more effectively targeted preventions and interventions to reduce loss of life to suicide.

## Introduction

**Fig. 1:**
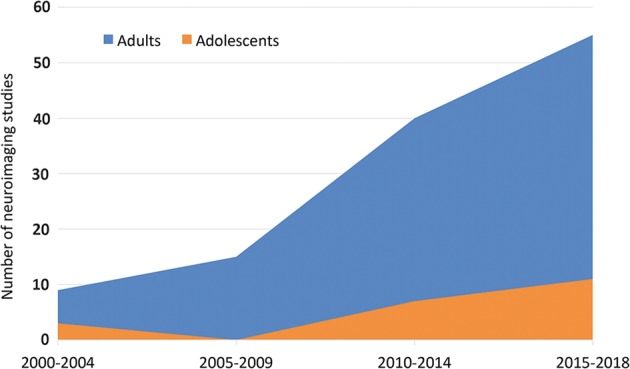
Number of neuroimaging studies on suicidal thoughts and behaviors published in the last 2 decades. The figure was based on the studies included in this review, calculated separately for studies only including adolescents and studies only including adults and divided into separate 4-year time bins for publication date

Around 1 million people die by suicide annually [1]. Globally, suicide is the tenth leading cause of death for all ages and the second leading cause of death among young people aged 15–29 years [1]. In 2013, it was estimated that 9.3 million adults 18 years and older in the United States had suicidal thoughts and 1.3 million attempted suicide [2]. In addition, a 2011 report estimated that 13% of adolescents planned a suicide attempt (SA) in the previous year and 8% attempted suicide [3]. Unfortunately, suicide death rates have continued to rise. For example, since 1999, rates in the United States have increased by 30% [4]. The predictive value of currently identified nonbiological risk factors for suicide is limited [5], and a reliable biological risk marker has yet to be identified. In order to prevent suicide more effectively, there is an urgent need to better understand the mechanisms that confer increased risk for suicidal thoughts and behaviors (STBs), and to identify biological markers of risk to generate more targeted successful prevention strategies and monitor responses to them. In the last decade, and especially in the last 5 years, there has been exponential growth in the number of neuroimaging studies reporting structural and functional brain circuitry correlates of STBs (Fig. 1). In the last 10 years, a number of excellent reviews on aspects of this research have emerged [6–10]. Here we review research across structural, functional, and neurochemical neuroimaging modalities, providing a narrative review of 131 neuroimaging studies with a focus on the most researched brain circuitries and findings that converge across studies.

## Methods

A search was performed in PubMed for original research articles published before March 12, 2018. The following terms were used: “MRI,” “SPECT,” “PET,” “magnetic resonance imaging,” “positron emission tomography (PET),” “single-photon emission computed tomography,” “DTI,” “diffusion tensor imaging,” “diffusion weighted imaging,” “neuroimaging,” “functional MRI (fMRI),” “functional magnetic resonance imaging,” and “spectroscopy” (separated by OR) in combination with the terms “suicide,” “suicidal,” and “suicidality” (separated by OR). We selected articles that: (i) were published in a peer-reviewed journal in English and (ii) included groups with suicidal ideation (SI) and/or history of SA. Of note, nonsuicidal self-injury (NSSI) was not included in this review, and studies that did not differentiate NSSI from suicidal behaviors were excluded, because these can be differentiated on the basis of intention, frequency, and lethality and may have partly distinct underlying mechanisms [11].

## Results

We identified 131 unique articles meeting review criteria (Supplementary Tables 1–3). The populations studied reflect reports that the majority of people with STBs have a diagnosable mental illness. Major depressive disorder (MDD) or bipolar disorder (BD) account for over half of suicide deaths [12]. After mood disorders and borderline personality disorder (BPD), the prevalence of suicide deaths is highest among people with substance use disorders and schizophrenia (SZ), followed by posttraumatic stress disorder (PTSD) and anxiety disorders [12]. The mental disorders researched varied by study, with each study typically including a single disorder, and the majority of studies conducted in individuals with mood disorders. Most studies compared people with a mental disorder and a history of SA (suicide attempters, SAs) to people with a mental disorder and/or healthy controls (HCs) without a history of attempt. Fewer studies focused on SI. Most studies employed a cross-sectional design and a single structural or functional imaging modality. The majority were conducted with adults; only a small proportion examined  adolescents (Fig. 1). A subset of studies provided preliminary findings of associations between neuroimaging measures and key risk factors for suicide, e.g., medical lethality of prior attempts, emotion dysregulation, anhedonia, impulsiveness, and reduced cognitive control (for reviews see [13–15]).

**Fig. 2:**
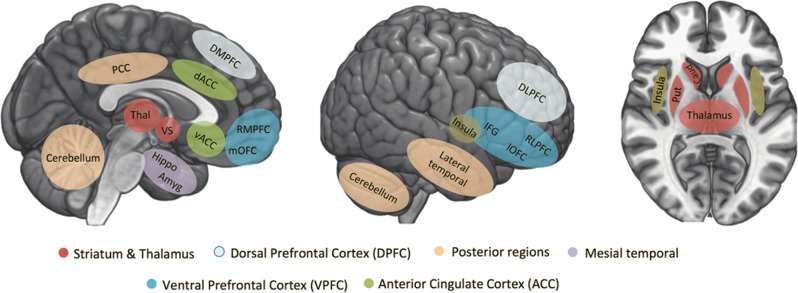
Overview of brain regions included in this review. These brain regions have been most reported in neuroimaging studies investigating structural, functional, and molecular brain alterations associated with suicidal thoughts and behaviors, with a subset of regions grouped more broadly into ventral prefrontal cortex, dorsal prefrontal cortex, insula, mesial temporal, subcortical, and posterior regions. DMPFC dorsomedial prefrontal cortex, dACC dorsal anterior cingulate cortex, RMPFC rostromedial prefrontal cortex, mOFC medial orbitofrontal cortex, vACC ventral anterior cingulate cortex, PCC posterior cingulate cortex, Thal thalamus, VS ventral striatum, Hippo hippocampus, Amyg amygdala, DLPFC dorsolateral prefrontal cortex, RLPFC rostrolateral prefrontal cortex, IFG inferior frontal gyrus, lOFC lateral orbitofrontal cortex, Put putamen, Caud caudate

Despite modest sample sizes of studies, and the heterogeneity of their clinical samples and neuroimaging acquisition and analysis methods, converging evidence is emerging to support roles for specific brain regions/circuitries in STBs. These are particularly in cortico-striatolimbic systems that subserve emotion and impulse regulation and include prefrontal, cingulate, and insula cortices, amygdala, hippocampus, thalamus, and striatum regions (Supplementary Tables 1–3). Within the prefrontal cortex (PFC), studies vary widely in the selection of regions studied, with regions of interest (ROIs) often including overlapping regions that encompass ventral and dorsal, medial as well as lateral, PFC. Thus, while we identify the importance for future study of specific PFC subregions, given their differing connectivity, cellular and molecular features and functions, we discuss the PFC grouped broadly into the ventral PFC (VPFC; divided into medial and lateral portions), which has the highest concentration of reported findings, the dorsal PFC (DPFC; divided into lateral and medial portions), and the anterior cingulate cortex (ACC). We also discuss findings in the insula, and mesial temporal (hippocampus, amygdala), subcortical (basal ganglia, thalamus), and posterior regions (posterior cingulate cortex (PCC), lateral temporal lobes and cerebellum). See Fig. 2 for definitions of the brain regions. Since the VPFC, DPFC, ACC, insula, mesial temporal, basal ganglia, thalamus, and posterior regions have been studied most frequently in relation to STBs and because most converging evidence exist for the involvement of these regions, we specifically focus our review on findings within these brain areas, as well as in the connections between them. However, additional studies and positive and negative findings not discussed below can be found in Supplementary Tables 1–3.

We focus our discussion below on findings that are primarily derived from comparisons of individuals with a mental disorder and STBs vs individuals with a mental disorder without STBs (diagnostic controls (DCs)), rather than comparisons with HCs, unless otherwise specified. Findings based on a comparison between DCs and individuals with the same mental disorder plus STBs are more commonly reported in the literature and are more likely to reflect specific effects of STBs in that disorder, whereas comparisons with HCs may include more general effects of having a mental disorder. Below we first detail structural, functional, and neurochemical findings within regions and end each section on a region with a summary. We then follow with a section devoted to studies of connectivity among regions within major implicated brain systems.

### Ventral PFC

The lateral VPFC (VLPFC) refers to the inferior lateral areas of the frontal cortex encompassing lateral orbitofrontal cortex (OFC, Brodmann area (BA)47, lateral BA11), inferior frontal gyrus (IFG, BAs 44, 45), and lateral aspects of the rostral PFC (RLPFC, lateral BA10) (Fig. 2). The VLPFC plays a key role in cognitive control, including response inhibition, and is activated when behavioral responses are modulated in response to the emotional or motivational context [16, 17]. The medial VPFC (VMPFC) refers to the medial OFC (medial BA11) and medial aspects of the rostral PFC (RMPFC, medial BA10) (Fig. 2). The VMPFC has a well-established role in self-reflection [18], appraisal of internally generated emotions (both positive and negative) [16, 19], appraisal of past and imagined future events, and reward processing [20, 21]. Structural, functional, and neurochemical alterations in these regions have been associated with maladaptive strategies for regulation of negative affect (e.g., rumination), negative self‐referential thinking [22, 23], and diminished positive affect (e.g., anhedonia) [24]. Evidence is mounting that emotion dysregulation has a central role in the generation of STBs. This includes elevations in negative, and blunting in positive, subjective emotions, self-referential thoughts, and responses to valenced stimuli [9]. These alterations are thought to contribute to key clinical risk symptoms for STBs including depression, anxiety, rumination, guilt, reduced self-esteem, helplessness, anhedonia, and hopelessness [22–30].

#### VLPFC

Structural MRI studies have consistently shown lower gray matter volumes of the VLPFC in adult SAs, including SAs with MDD [31–33], BD [34, 35], and schizoaffective disorder (SZA) [36]. Findings of lateral OFC volume decreases, extending to medial OFC, in adolescent and young adult SAs with BD [35], suggest these may be early differences. Lower VLPFC thickness, but not volume, is one of the rare findings related to SI in adults with MDD [37], suggesting the VLPFC may be involved not only in suicide behavior but also in the ideation that may generate it.

Gray matter volume decreases in VLPFC were also associated with high lethality of prior SA in MDD [24] and BPD [35, 36]. This converges with longstanding findings from both postmortem studies demonstrating VPFC (ROIs including both VLPFC and VMPFC) differences in people who died by suicide [38], and PET studies of high lethality or high intent SAs, showing VPFC (VLPFC and VMPFC) alterations in serotonin (5-HT) synthesis, transporters and 5-HT1a receptors [39, 40] (Supplementary Table 2). Lower 5-HT1a binding in the OFC was associated with interim SI during a 2-year follow-up in adults with MDD and past attempts [41]. Although conflicting findings exist [42–45], these data provide some consistent findings of a location and a potential mechanism, i.e., lateral extending to VMPFC serotonergic dysfunction, as a potential biomarker of risk for high lethality STBs. There have been few molecular imaging studies of other neurotransmitter systems and neurochemicals implicated in STBs.

fMRI studies performed while participants conducted specific behavioral tasks provide evidence that STBs are associated with VLPFC functional abnormalities in response to emotional and other hedonically valenced stimuli. Increased activation of the IFG and lateral and medial OFC while viewing angry (but not happy, sad, or neutral) faces was reported in adult SAs with MDD [46, 47]. Higher IFG activation in response to angry faces was also associated with poorer attempt planning and higher impulsivity in adult SAs with MDD [48]. Furthermore, young adult SAs with BPD displayed higher lateral OFC activation while instructed to experience and regulate negative autobiographical memories [49]. Increased activation in a region of interest that included both the medial and lateral OFC was also seen in response to winning a reward [46] in adult SAs with MDD.

The IFG also plays a critical role in cognitive control and response inhibition [50]. During the performance of a continuous performance task, higher IFG, RLPFC, and lateral OFC responses were associated with both attempts and SI in adults with mood disorders with psychotic features, in the absence of task performance differences [51]. Higher lateral OFC was also reported during error trials in a response inhibition task in veterans with SI [52]. In contrast, a second study in adult SAs with SZ using the same continuous performance task showed that reduced activation in a cluster encompassing the RLPFC and IFG, extending to the VMPFC and ventral ACC, was associated with SI but did not further distinguish between ideators with and without a history of attempt [53].

#### VMPFC

In addition to the structural and PET study findings reported for the VLPFC above that extended to the VMPFC or that were based on an ROI including both VLPFC and VMPFC, lower VMPFC cortical thickness was also associated with greater motor impulsivity in adolescent SAs with MDD [54]. As cortical thickness and surface area contributions to volume are thought to be genetically independent [55] and result from different neurobiological processes [56], it is important to examine these separately in studies of STBs. However, the majority of studies have either not examined thickness and surface area separately from volume or examined only thickness without examination of surface area in SAs with MDD [31, 57]. Cortical thickness is thought to be influenced by the number and the size of cells within a column, packing density, as well as by the number of connections and the extent of their myelination, while cortical surface area is driven by the number ontogenetic columns that run perpendicular to the surface of brain [58].

Functionally, in addition to higher activation in the lateral and medial OFC in response to angry faces and to winning a reward in adult SAs with MDD [46] as reported above, a recent study using machine learning to investigate adolescent SAs (with and without SI) showed that the medial VMPFC was among the most discriminating regions, within a multivariate pattern of fMRI brain activation in response to actively thinking about life- and death-related concepts, for distinguishing between adolescent suicidal ideators with and without a history of attempt, although in a very small sample size [59].

#### Summary

Structural neuroimaging studies have consistently shown that alterations in the VLPFC and VMPFC are implicated in SAs across a range of mental disorders and age ranges. Reduced VLPFC volumes were also associated with lethality of attempts, potentially mediated by serotonergic dysfunction, although findings of serotonergic dysregulation remain inconsistent. The involvement of structural and functional VLPFC and VMPFC alterations in SI remains understudied. Viewing and regulating negative emotions and motivationally valenced stimuli has been linked to increased activation of the lateral and medial OFC in adult (including young adults) SAs with MDD and BPD, and associated with poorer attempt planning and higher impulsivity. Finally, higher VLPFC activity, including in the IFG, RLPFC, and lateral OFC, during cognitive control and response inhibition in relation to SAs and SI in adults with mood disorders has been reported across a number of studies. These increased activations may, in the absence of task performance differences, reflect a need for greater engagement of these regions for reaching similar performance in these individuals with STBs. In contrast, adult SAs with SZ showed reduced activation in these regions during cognitive control, which may suggest that there are some differences in the neural signatures of STBs between mood and psychotic disorders, which will be an important direction for future study.

### Dorsal PFC

The DPFC can be broadly divided into dorsolateral (DLPFC) and dorsomedial PFC (DMPFC). The DMPFC and the DLPFC together support top-down control of emotions and behaviors [17], cognitive flexibility, and complex decision-making [60]. Deficits in these processes are thought to have an important role in STBs, particularly in the transition from SI to behavior, as the threshold to acting is lowered by decreased top-down behavioral inhibition, and diminished flexibility in generating alternate and more adaptive behavioral choices [15, 61]. Neuroimaging evidence suggests that the DMPFC (medial portions of BAs 8 and 9) is robustly recruited during tasks that require mental state inference [62, 63]. The DMPFC is further involved in tracking decision conflict and reinforcement history [64], as well as in emotion regulation [65]. The DLPFC (BA46, lateral BA9) is involved in the conscious active control of planned behavior and cognition, as well as working memory [66]. Access of the DLPFC to memory processing in hippocampal regions is shared by the rostrolateral PFC (lateral frontal pole BA10), which has been implicated in meta-cognitive awareness [67, 68].

#### DLPFC

Although the amount of evidence to date has been less than in VPFC, accumulating findings also support a role for the DLPFC in STBs. Structural MRI studies support lower volume in DLPFC in adult SAs across MDD [31, 32, 69] and BD [32, 34]. Studies showed lower DLPFC thickness in adult SAs with MDD [57] and SZ [70], but have not examined cortical surface area. In addition, lower DLPFC volume was associated with attempt lethality in mood and psychotic disorders [31, 36]. Higher baseline 5-HT1a receptor binding potential in the DLPFC was also associated with higher lethality of future attempts and SI during a 2-year follow-up [41].

Functionally, decreased lateral (and medial) DPFC activation when ten adults with self-reported depression listened to their own narrative of their attempt was reported in a study in which imaging was conducted close to the time of the attempts (1–4 weeks prior [71]). A study of adolescents with a history of SI showing lower right DLPFC activation during passive viewing of negative emotional scenes suggests that DLPFC decreases during processing of negative emotional stimuli might be present early in the course of SI [72]. Higher right DLPFC engagement was observed during regulation of responses to negative emotional scenes in the same adolescents, suggesting the direction of DLPFC differences depends on the specific task requirements (passive viewing vs regulating) [72]. Another study in adolescent SAs with MDD suggests that the direction may also relate to the specific emotion, as passive viewing of angry faces, but not happy faces, elicited higher DLPFC responses [73], perhaps due to the high sensitivity to criticism and social rejection previously reported in individuals with STBs [74, 75].

The DLPFC’s critical role in decision-making in the context of evaluating the motivational value of choices [76] may be especially relevant to STBs. Blunted DLPFC activation was observed when evaluating risky vs safe options in adult SAs with MDD [46] and when evaluating lower immediate rewards vs larger delayed rewards in older adults with MDD and well-planned SA [77]. This is in line with a behavioral study of older adult SAs with MDD showing an association between lower levels of delay discounting (or impulsive decision-making) and better planning in individuals with SA [78]. Increased DLPFC activation has also been observed in adults with STBs across a range of “cold” cognitive control tasks, especially those requiring inhibition of automatic response tendencies. These included paradigms such as continuous performance, stop signal, go-no-go and stroop tasks studied in adult SAs with MDD or BD with psychotic features [51], SZ [79], and ideators with SZ [80] or PTSD and MDD in veterans [52]. Elevated activation in DLPFC was observed in suicidal ideators with past attempts compared with ideators without attempts while performing a continuous performance task [51]. Single-photon emission tomography (SPECT) and PET studies have shown lower resting regional cerebral blood flow (rCBF) and glucose metabolic rates (rCMRglu) in the DLPFC in adult SAs with mood disorders [81, 82]. Moreover, lower DLPFC rCMRglu was associated with higher lethality of attempt [83] and with SI with a plan vs ideation without a specific plan [84] in adults with MDD.

#### DMPFC

Structural MRI studies also support lower volume in DMPFC in adult SAs with MDD [31, 69] and BD [32, 34]. Although less studied than DLPFC in functional neuroimaging, lower DMPFC was reported in the study in which adults with depression listened to their own narrative of their recent attempt, which was especially pronounced during mental pain aspects of the narrative [71]. Furthermore, decreased activation was also found in DMPFC during viewing of angry faces in adult SAs with MDD [47].

#### Summary

Structural alterations in both the DLPFC and DMPFC have been consistently observed in adults across mental disorders and structural alterations in the DLPFC have been associated with attempt lethality. This latter finding, together with findings of serotonergic dysfunction in the DLPFC being associated with higher lethality of future attempts during a 2-year follow-up, implicates DLPFC structure and serotonergic system functioning in STB risk. With regard to DLPFC and DMPFC functioning, there is convergence in showing differences related to STBs during processing of negative emotional stimuli, although the direction of effects (activation increases vs decreases) in the DLPFC differed across studies, with contributions to the differences unclear as the studies differed across multiple variables. Elevated activation while performing cognitive control tasks (in the absence of performance differences) together with lower resting rCBF and glucose metabolic rates in the DLPFC could suggest that increased DPFC may be recruited for reaching similar task performance perhaps due to lower baseline levels of activation in the DPFC regions. Findings from one study suggests that functional DLPFC alterations during cognitive control can discriminate between suicidal ideators with past attempts and ideators without attempts. Moreover, blunted DLPFC activation when evaluating the value of different decision options may also represent a risk marker for SAs, and especially for well-planned attempts. Well-planned vs impulsive SAs have been suggested to be different phenotypes [85]. From the papers reviewed, there was a greater concentration of findings in the VPFC in impulsive SAs and in DPFC in planful SAs. Therefore, we speculate that the relative ventral vs. dorsal localization of the PFC abnormalities may contribute to the differing phenotypes.

### Anterior cingulate cortex

The ventral ACC consists of BA25 and ventral BA32 sub- and pregenual to the corpus callosum and plays a critical role in valuation and control of autonomic viscero-sensory signals, the modulation of physiological responses to stress, and the appraisal of internal feelings [16]. The dorsal ACC (dACC) (dorsal BAs 24 and 32) plays an important role in the appraisal of actions (and adaptively adjusting behavior as a consequence) and reward-based decision-making [16].

Structural MRI studies support lower volume in both ventral and dACC in adult SAs with MDD [33, 57] and BD [34], which was related to a higher number of attempts in adolescents with BPD and MDD [86] and to higher lethality of attempts in adults with BPD [87] and psychotic BD [36]. Lower dACC rCMRglu was associated with higher lethality of attempts [83]. In addition, lower 5-HT1a binding in the ACC was associated with interim SI during a 2-year follow-up in adults with MDD and past attempts [41]. A few studies that investigated neurotransmitter systems other than 5-HT implicated the ACC in relation to SI. For example, a positive association was shown between SI and ACC monoamine oxidase-A density in adults with BPD [88]. A relation between SI and increased ACC neuroinflammation (as assessed by translocator protein (TSPO) availability) was reported in adults with MDD [89]. Furthermore, dACC gamma-aminobutyric acid (GABA) concentrations were lower in adult female SA + SI compared with clinical controls without SA or SI, however, this effect was no longer significant after correcting for age [90].

Viewing of angry faces elicited higher dACC responses in adolescent SAs with MDD [73], perhaps due to the high sensitivity to criticism and social rejection previously reported in individuals with STBs [74, 75]. In contrast, decreased activation was found in the ACC (ROI capturing both ventral and dACC) during the viewing of sad faces in adult SAs with MDD [46]. With regard to positive stimuli, blunted ventral ACC responses were found during the anticipation of reward in adult [91], including elderly [92], SAs with MDD. Elevated responses in the ventral ACC have also been reported in relation to positive stimuli. For example, higher activation in the ventral ACC was seen in response to happy facial expressions [47] and in response to actual winning [46] (in contrast to blunted responses during reward anticipation) in adult SAs with MDD.

#### Summary

The ACC has mostly been studied in relation to emotional processing. Although various studies have observed dorsal and ventral ACC activation alterations in adolescents and adults with MDD and SAs, the direction of alterations seem to be complex and dependent on task condition and stimulus type (positive vs. negative). One could perhaps interpret the findings of increased dorsal and ventral activation in response to angry faces and to positive stimuli, together with blunted ventral ACC activation during reward anticipation, as negative biases, as they may reflect reduced reward anticipation (anticipation phase) vs. increased activation in response to negative stimuli and in relation to positive prediction errors in response to positive stimuli (outcome phase). The finding of a positive relation between SI and ACC neuroinflammation, together with findings of increased inflammatory markers in the ACC in postmortem studies of people who died by suicide [47], and in the blood and cerebrospinal fluid of people with SI and a history of violent or high intent attempts [48, 49], suggests that neuroinflammation in the ACC may constitute a promising target for future studies of STBs.

### Insula

The insular cortex is a key hub in emotional processing with connectivity to the PFC, particularly VPFC, as well as mesial temporal structures [93]. The insula plays an important role in interoceptive awareness for positive and negative internal states [94], including emotional and other types of pain, and understanding and sharing of other people’s emotional states [95, 96]. Only in more recent studies has insula structure and function and related behavior been investigated for its role in STBs. For example, on a behavioral level, interoceptive deficits have been reported among SAs compared with individuals who only thought about or planned suicide among general psychiatric outpatient adults [97] and predicted SI severity at 6-month follow-up in community adolescents [98].

Smaller insula volume has been reported in adult SAs with BPD [99], in a combined group with SZ/SZA/psychotic BD [36] and elderly with MDD [69]. Lower insula thickness was observed in adults in relation to SA in SZ [70] and SI in MDD [37]. Smaller insula volume was associated with higher attempt lethality and lower impulsivity in BPD [87, 99]. In contrast, larger insula volumes were reported in relation to attempt lethality in adults with BD [100]. It is possible that the type of insula differences relate to specific characteristics of the high lethality attempters, since larger insula volumes were also found in association with higher lifetime history of aggression in BPD [87]. Some findings in the PFC noted above in MDD extended to the insula, including associations between baseline 5-HT1a binding potential with SI and lethality of future attempts within a 2-year follow-up period [41] and of increased neuroinflammation (TPSO availability) [89].

SPECT research showed higher insula rCBF in adult SAs with MDD [101] at rest and higher insula fMRI activation was found in adults with MDD or BD with psychotic features during a cognitive control task with insula activity related to higher intensity of SI [51]. Higher insula fMRI activation was also associated with lower subjective value of gain and loss in adult SAs with MDD [91]. Lower activation in the posterior insula during social exclusion was found in adult SAs with MDD or BD, which was suggested to indicate a higher tolerance to pain via repeated exposure to painful and provocative experiences in subjects vulnerable to suicide [102].

#### Summary

Smaller insula volume has been associated with SAs and lower impulsivity in adults across various mental disorders, whilst, both smaller and larger insula volumes have been associated with higher attempt lethality. fMRI studies found higher insular activation during reward processing and cognitive control in adult SAs with MDD, while lower insula activation was associated with a higher tolerance to social pain in adult SAs with MDD or BD. Thus, there is preliminary evidence for an involvement of insular structural and functional alterations in SI and SAs. However, since very few studies have focussed on the insula and that both decreases and increases in insula alterations have been reported, more research is needed to elucidate the role of the insula in STBs. Interestingly, immune challenges activate interoceptive brain pathways (including the insula), triggering alterations in mood and cognition, motivation, and neurovegetative processes [103]. Together with preliminary evidence of increased neuroinflammation in the insula related to SI, this suggests that the insula may be an important region for future studies of neuroinflammation and STBs.

### Amygdala and hippocampus

Due to their roles in processing of emotion, emotional memory and stress response [104–107], the mesial temporal amygdala, hippocampus, and entorhinal cortex (BA28, within the adjacent parahippocampal gyrus) are also thought to be involved in STBs. However, findings reported have been inconsistent. Larger amygdala volumes were reported in adult SAs with MDD [108] and SZ [109], but more studies have not detected significant amygdala findings [32, 37, 99, 100, 110–112]. Smaller hippocampal volumes were reported in adult SAs with MDD [113] and adolescent and young adult SAs with BD [35], and one study reported a smaller parahippocampal gyrus [114]. However, more studies have not detected associations of hippocampal or parahippocampal volume with STBs [32–34, 37, 100, 108–111]. While difficulties detecting differences in these small mesial temporal structures may relate to imaging methods used, it may be that mesial temporal alterations are only present in specific subgroups of people with STBs. For example, high lethality attempts were associated with smaller volumes of the hippocampus and parahippocampal gyrus in adult SAs with BPD [87, 99]. In addition, amygdala and hippocampal volumes were negatively associated with impulsivity in individuals with low lethality attempts [87], and amygdala volume was positively associated with self-aggression in SAs with SZ [109].

PET studies have shown preliminary evidence for a role of serotonergic alterations in the amygdala and hippocampus in STBs. Increased hippocampus 5-HT2a receptor binding and 5-HT release was observed in adult SAs with BPD [115] and in adults with MDD and high lethality attempts [39], respectively, compared with HCs. Recently, baseline 5-HT1a receptor binding in the amygdala, hippocampus, and parahippocampal gyrus was associated with higher SI during a 2-year follow-up in adults with MDD [41].

Few fMRI studies have focused on mesial temporal ROIs. An activation study focusing on the amygdala found no association between SI and amygdala functioning during emotion processing in ten ideators [116]. Increases were observed during autobiographical recall of mental pain experienced during an ideator’s own attempt in right parahippocampal gyrus vs suicide action in left hippocampus [71]. In contrast, parahippocampal gyrus activation was blunted in adult SAs with MDD choosing between a smaller immediate reward vs. a larger but delayed reward, especially when the two rewards were more than 1 year apart [77]. Given the role of the parahippocampal gyrus in prospection [117], its blunted response to prospects with longer vs. shorter delays may represent a neural substrate of impaired prospection in SAs [118–120], potentially undermining the deterrents and the generation of alternative solutions during a suicidal crisis.

#### Summary

Although structural alterations in the amygdala and the hippocampus have been consistently implicated in mental disorders [121–123], the majority of studies reviewed do not report structural alterations in these regions in relation to STBs. These mixed findings could perhaps be explained if additional involvement of the amygdala and hippocampus in STBs beyond their role in mental disorders is subtle with small effects only apparent in studies with very large sample sizes. This is consistent with a post hoc power analysis based on observed effect sizes in the largest study on subcortical volumes in STBs to date [110]. Alternatively, mesial temporal structural alterations may only be present in specific subgroups of people with STBs. Preliminary evidence suggests serotonergic alterations in the amygdala and hippocampus are linked to SAs as well as SI across mental disorders, and altered functioning in these regions is related to increased autobiographical recall of mental pain, blunted immediate reward processing, and impaired prospection in individuals who made SAs, although molecular and functional studies focussing on these regions are still scarce.

### Striatum and thalamus

The ventral striatum includes the nucleus accumbens and ventral parts of the putamen and caudate [124] and is a core region of the reward network [125]. Dorsal striatum, including dorsal caudate and putamen, functions include initiating action, inhibitory control, and stimulus-response learning [126]. The striatum projects to the frontal lobe via the thalamus [124], which is also involved in sensory processing [127].

Lower caudate and putamen volumes have been reported in adult SAs with MDD in comparison to MDD nonattempters [33, 128] and HCs [129], and putamen volumes were negatively associated with impulsivity [128]. Lower putamen binding of the serotonin transporter (5-HTT) was also reported in adult SAs with MDD compared with HCs [130], and was negatively associated with impulsivity [131]. However, striatal 5-HT binding was positively associated with SI in adult SAs with MDD [132]. With regard to the thalamus, higher volumes were reported in veterans with traumatic brain injury and SAs [133] and higher 5-HT synthesis was reported in adult SAs with a mix of psychiatric diagnoses [39]. However, other studies, including the largest study to date of individuals with STBs (*N* = 451), have not detected associations of striatum and thalamus volume with STBs [32, 37, 110].

Few fMRI studies have examined striatum and thalamus regions. Positive correlations were observed between the intensity of past SI and dorsal striatum responses during cognitive control in adults with MDD or BD with psychotic features [51]. Lower putamen activation in adults with BD during a motor task was associated with higher SI [134]. Higher thalamus activation was observed when viewing knives (vs. landscapes) [135] and higher thalamus activation during response inhibition (go-no-go task) was associated with higher levels of mental pain and suicide intent [136] in adults with SAs and MDD.

#### Summary

Mixed findings were reported for the involvement of structural alterations in the striatum and thalamus in relation to STBs, with the largest sample to date showing no associations in people with MDD. Increased dorsal striatum responses were found during cognitive control in the absence of performance differences in individuals with SI, suggesting greater engagement of this region to reach similar levels of cognitive control. Higher thalamus activations were reported during emotion processing and inhibition and associated with SI in adult SA with MDD. Structural and 5-HTT alterations in the dorsal striatum specifically linked to impulsivity in adult MDD with SA converge with findings of functional alterations in the dorsal striatum in relation to diminished cognitive and affective control associated with SI. Of note, alterations in the ventral striatum have been proposed to underlie reduced reward anticipation and anhedonia in individuals with STBs [137]. No studies reported ventral striatal activity, however, ventral striatal connectivity findings during reward processing and during rest are discussed in the “Structural and functional connectivity” section below.

### Posterior structures

The temporal association cortices are involved in the perceptual processing of faces and other complex object features [138, 139], auditory information and language [140]. Consistent with this, structural MRI findings in lateral temporal cortex were observed in adult SAs with SZ/SZA/psychotic BD and other disorders in which psychotic misperceptions can be observed. Lower middle and superior temporal gyrus volume was found in adult SAs with primary psychotic disorders [36, 141] and BPD [99], and lower thickness of middle and superior temporal gyri were observed in adult SAs with SZ [70]. Lower middle and superior temporal volumes were also associated with high lethality attempts in adults SAs with BPD [87, 99]. Serotonin system studies have yielded various results including lower 5-HTT temporal binding associated with higher impulsivity in adult SAs with different mental disorders [131], and higher baseline 5-HT1a temporal lobe receptor binding in adults SAs with MDD [41] associated with higher levels of SI at 2-year follow-up. fMRI studies also suggest a role of the lateral temporal lobe in emotion processing in STBs. Specifically, adolescent SAs with MDD showed enhanced right middle temporal gyrus activation during passive viewing of angry, happy, and neutral facial expressions [73] and during recall and reimagination of suicidal episodes in adult SAs with MDD [71]. SI was associated with increased superior temporal activation during error processing in veterans with traumatic brain injury [52]. In contrast, lower perfusion in these temporal regions during rest (measured by rCBF) was reported in adults with MDD and SA [81, 142].

A few studies implicate other posterior brain regions including the PCC and cerebellum in STBs (Supplementary Tables 1–3). The PCC is implicated in psychological processes that may be linked to STBs, including controlling the vividness of negative mental imagery [143] and enhancing self-referential processing [144]. Lower PCC gray matter volume was found in adult SAs with MDD [145]. Decreased PCC activation was observed during cognitive control in adult SAs with psychotic mood disorders [51] and during self-referential processing in adolescent ideators with MDD [146], although adult SAs with depressive disorders, compared with HCs, showed increased PCC response when viewing knives [135].

The cerebellum is increasingly recognized for its involvement in emotional processes [147, 148]. Lower volumes of the cerebellum were reported in adult and adolescent SAs with MDD or BD [35, 69, 145, 149]. Functionally, while adult SAs with MDD showed increased cerebellum activation during recall and reimagination of their own suicidal episode [71] and while viewing angry faces, they showed decreased activation while viewing happy faces [47]. Decreased activation was also observed while passively viewing negative emotional pictures in adolescents with a history of SI [72]. Finally, ketamine-induced reductions in SI were associated with increases in rCMRglu including in cerebellum [150].

#### Summary

Lower middle and superior temporal gyri volumes have been reported in six studies across a range of mental disorders, and were related to high lethality attempts and higher impulsivity. Serotonergic alterations in these regions have not been extensively investigated and directions of reported effects are mixed. Increased activation in middle and superior temporal gyri have also been reported in adults and adolescents with SA and MDD, especially in relation to emotion processing. Preliminary evidence suggests a role for the PCC and cerebellum in STBs, especially in relation to self-referential and (autobiographical) emotion processing, but studies investigating these regions remain scarce. Of interest, one study suggests that ketamine-induced changes in SI are associated with ketamaine-induced increases in cerebellum rCMRglu. This medication-related finding is a potential lead in understanding brain mechanisms that may be helpful targets for suicide prevention interventions, but requires replication.

### Structural and functional connectivity

Disturbances in the structure and function within brain regions can result in alterations in brain networks, including the ability of brain regions to coordinate their activity in a system. System dysfunction can also result from abnormalities in the connections between regions. Increasingly, abnormalities in the structural and functional connections between brain regions within larger-scale brain networks have been reported in studies of STBs.

Connections of the VMPFC with other cortical midline structures (PCC, precuneus) and temporal and parietal regions are implicated in the brain’s default mode network and play an important role in self-referential processes, social cognition, autobiographical memory, and prospective imagery [117]. Lower resting functional connectivity was reported in the default mode network in adolescents with MDD and SI [151]. These findings are in line with findings of lower resting functional connectivity of rostral ACC with medial OFC, precuneus, and temporal pole in adults with MDD and SI [152] and within the precuneus in young adult SAs with MDD [153]. Structurally, lower fractional anisotropy (FA; thought to reflect the structural integrity of white matter and the neuronal connections it contains [154]) in the VMPFC was reported in adult SAs with BD, which was associated with higher motor impulsivity [155]. Lower FA was also reported in the ventral cingulum (connecting posterior and temporal default mode network regions) in adults with MDD and SI [37]. Lower FA of the corpus callosum genu, which provides connections of interhemispheric anterior default mode network regions, was associated with a higher number of SAs in BD, MDD, and BPD [156, 157].

The limbic network includes the amygdala, medial and lateral OFC, medial temporal regions, thalamus, and basal ganglia and is involved in emotional and autonomic processes [158]. Using task fMRI, lower amygdala-VMPFC/rostral PFC connectivity was found in adolescent and young adult SAs with BD while viewing happy and neutral facial expression, and associated with higher lethality of attempts and current SI [35]. During rest, greater amygdala connectivity with lateral OFC, insula, and middle temporal gyrus was found in adult SAs with MDD, with greater amygdala–parahippocampus connectivity associated with SI [159]. These functional connectivity alterations are in line with lower FA in the uncinate fasciculus, which provides major amygdala connections, in adolescents SAs with BD [35].

The medial OFC together with ventral striatum and the ventral tegmental area form core hubs of a reward network, with additional limbic regions, DLPFC and dACC forming a wider reward network subserving reward-related memory and evaluation [125]. An fMRI study investigating connectivity during reward processing showed a positive correlation between SI and connectivity of the left ventral striatum with dACC, DMPFC, and DLPFC during loss trials in adults with MDD [160]. Using resting state fMRI, Kim et al. [161] found reduced connectivity in a circuitry resembling this reward network, including the OFC, striatum, and thalamus, in adults with MDD and recent (past month) SI. This is in line with findings of lower structural connectivity between VPFC/OFC and striatal regions in adults with MDD and SI (33% also had a prior SA) [162], and between the ACC and OFC (as measured by graph theory) in adult SAs with MDD [163]. Lower FA was also reported in the anterior limb of the internal capsule (connecting striatal and thalamic regions with the PFC) in adults with MDD and attempts [164, 165].

The left and right DLPFC and DMPFC together with parietal regions comprise a network that plays a key role in cognitive control of thought, emotion, and behavior (executive control network [166]). Lower executive control network coherence during rest has been associated with both lifetime SI and past attempts in adolescents with MDD [151], in line with findings of lower DLPFC resting state connectivity in young adult SAs with MDD associated with higher impulsivity [167] and reduced white matter integrity (FA) in the DMPFC in adult SAs with MDD [168].

Alterations in dACC connectivity have been linked to STBs in the context of conflict monitoring, with different patterns of dACC connectivity associated with SI vs SAs in adults with recent-onset SZ [169]. That is, the presence of lifetime SI was positively associated with magnitude of functional connectivity of dACC with the precuneus, a core hub of the default mode network. This may suggest a reduced capacity of the dACC to “switch off” default mode network activity associated with more internally focused attention, when activation of externally focused cognitive processing is required [169]. In contrast, history of SA was negatively associated with dACC connectivity with DPFC (BA9, 8, lateral BA10), VLPFC (BA45), PCC, parietal regions (BA7, 40) and superior and middle temporal gyri (BA22, 39, 40) [169]. These findings may suggest that SI and SAs have divergent bases in dACC connectivity with default mode network vs lateral PFC circuitries, respectively, in the context of monitoring conflict in adult SAs with recent-onset SZ. This is supported by findings of abnormal conflict-related dACC connectivity with VLPFC, OFC, insula, and striatum associated with SI intensity, but altered dACC connectivity with DLPFC and frontal motor regions associated with past SA in adults with MDD or BD with psychotic features [170]. In addition, decreased functional connectivity of the dACC with bilateral insula while viewing angry faces was also reported in adolescent SAs with MDD [73]. Connectivity between the dACC and insula plays an important role in detecting salient internal and external stimuli to guide behavior [171] and has been implicated in the anticipation of aversive experiences, especially in depressed individuals [172]. Reduced connectivity between the dACC and bilateral insula may indicate inefficient strategies to process the salience of, and select contextually appropriate behavioral responses to, negative emotional stimuli.

#### Summary

Emerging evidence suggests that resting state functional connectivity in the default mode network, and white matter tracts connecting regions within this network, play a role in both SI and SAs across mental disorders. Functional and structural connectivity alterations in the affective network have also been associated with SI and SAs, as well as lethality of attempts, both during emotion processing and during rest. Connectivity abnormalities in the reward network have mostly been examined in adults with MDD, and both structural and functional connectivity changes in regions of this network have been associated with SI and SAs in this group. Connectivity changes within and between regions implicated in the cognitive control network have been less extensively studied in relation to STBs, and the few studies conducted suggest a role of lower resting state functional connectivity within this network in STBs in adolescents and young adults with MDD. In addition, functional connectivity of dACC is implicated in STBs, but with divergent connectivity patterns related to SI vs SAs.

## Discussion

While the literature primarily includes cross-sectional studies with small sample sizes, differing clinical populations, and a wide range of imaging methods, there is emerging convergence in the brain regions implicated in STBs. Taken together with the recent increased momentum in studies on STBs (Fig. 1), it is very hopeful that significant advances in our understanding of brain mechanisms contributing to STBs are on the horizon. A critical frontier is to identify markers for elevated risk, especially short term risk in the transition from SI to attempt. While the majority of studies to date were on SAs, some studies specifically investigated associations with SI, and brain regions found to be associated with STBs have known roles in processes thought to contribute to STBs. Below, we briefly summarize the most convergent findings from the literature reviewed above, and propose directions for future neuroimaging studies on the neurobiology of STB.

### A tentative brain model of STBs

**Fig. 3:**
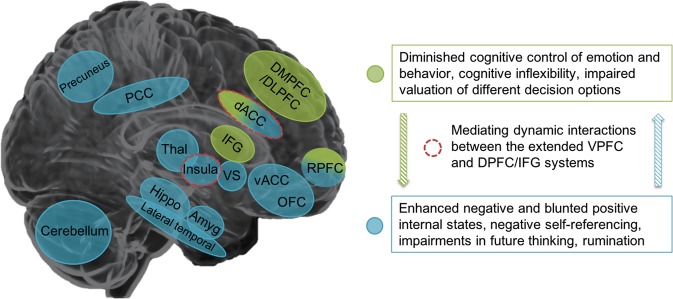
A tentative brain circuitry model of suicidal thoughts and behaviors. Medial VPFC (ventral ACC, OFC, and RPFC), insula, amygdala, hippocampus, lateral temporal regions, posterior midline structures (posterior cingulate cortex and precuneus), dACC, ventral striatum, thalamus, and cerebellum contribute to the generation of suicidal ideation through their roles in excessive negative and blunted positive internal states, negative self-referencing, impairments in future thinking and rumination. DPFC (DLPFC and DMPFC), IFG, RPFC and dACC alterations further exacerbate suicidal thoughts and facilitate suicide behaviors due to their involvement in diminished cognitive control of thought, emotion, and behavior and impairments in cognitive flexibility and valuation of different decision options. Alterations in bottom-up and top-down connections between these extended medial VPFC and DPFC/IFG systems may contribute to the transition from suicidal thoughts to behaviors. The dACC and insula may mediate this transition. Dashed lines indicate speculative associations that need further confirmation by future structural and functional connectivity studies. DMPFC dorsomedial prefrontal cortex, dACC dorsal anterior cingulate cortex, RPFC rostral prefrontal cortex, OFC orbitofrontal cortex, vACC ventral anterior cingulate cortex, PCC posterior cingulate cortex, Thal thalamus, VS ventral striatum, Hippo hippocampus, Amyg amygdala, DLPFC dorsolateral prefrontal cortex, IFG inferior frontal gyrus, Put putamen, Caud caudate

Figure 3 summarizes convergent findings emerging from the reviewed literature on brain alterations associated with STBs. Abnormalities in an extended VPFC system, including regions of the default mode, affective and reward networks such as the ACC, insula, medial and lateral OFC, RPFC, mesial temporal regions, ventral striatum and posterior structures (lateral temporal, PCC, precuneus, cerebellum), and in the connections among these regions, may be important in the excessive negative and blunted positive internal states that can stimulate SI. This is in line with the well-established role of this extended VPFC system in functions implicated in SI including appraisal of internally generated emotions, self-referential processing, recall of emotional episodic memories, imagining future positive and negative events, valuation of rewards, and integrating environmental stimuli to modulate subjective emotional states [16, 17, 66, 117, 125, 147]. A more lateral and dorsal system, including DMPFC, DLPFC, and dACC, together with the IFG and RPFC, may facilitate suicide behaviors through their role in cognitive control of thought, emotion and behavior as well as cognitive flexibility, complex decision-making (e.g., valuation of different decision options) and planning [16, 17]. A combination of VPFC and DPFC/IFG system disturbances may lead to very high risk circumstances in which SI may convert to lethal actions via decreased top-down inhibition of behavior or maladaptive, and inflexible decision-making and planning.

Alterations in the connections between these systems may contribute to the transition from suicidal thoughts to behaviors. For example, the dACC has connections to both the “emotional” ventral limbic system and the “cognitive” dorsal prefrontal system [173]. Consistent with this, findings suggest differential connectivity of the dACC in relation to SI vs. a history of attempt, with dACC connectivity with VPFC, insula, and striatal regions primarily associated with SI and dACC connectivity with DPFC regions associated with SA. The insula is also implicated in both SI and attempts and may perhaps be important for the transition from SI to attempt. Although the involvement of the insula in STBs has had little direct research focus, its critical involvement in interoceptive processing, detecting salient internal and external stimuli, experiencing emotion, and self-awareness [174–176] suggests an important role of the insula in SI. In addition, the insula is implicated in disconnection from bodily experiences, which in turn may lower the threshold to engaging in behaviors that harm the body (in line with the acquired capability theory [177]) thus suggesting a role of the insula in suicide behaviors. In line with important roles of dACC and insula circuitry (as part of the “salience network”) in mediating or switching between the extended VMPFC (default mode, affective, reward) systems and the DPFC/IFG (executive control) system [178–180], the dACC and insula may represent integral hubs that facilitate the transition from SI to attempt. However, this suggestion of the dACC and insula’s ability of mediating dynamic interactions between the VMPFC and DPFC/IFG systems and through these interactions playing a role in the transition from SI to attempt remains highly speculative and will need to be confirmed in future, preferably longitudinal, studies on the neurobiology of STB.

### Future directions

The literature reviewed underscores the need for future studies to include larger sample sizes and careful attention to developmental stages. In addition, studies employing longitudinal designs are critically needed to identify risk markers for future SAs in order to develop improved preventive strategies. Recent preliminary evidence, from a rare longitudinal study of adolescents and young adults with mood disorders over about 3 years, showed that those with future attempts had lower VPFC volume and decreased FA in VPFC and DPFC connections [181], suggesting that these may be potential predictors for STBs already present in adolescence. Longitudinal study over short time intervals are largely absent from the literature and are critically needed to assess proximal suicide risk.

Furthermore, studies focusing on identifying brain alterations that predict or fluctuate with changes in STBs following treatment could provide urgently needed biomarkers for response of STBs to existing interventions and could help develop novel treatments specifically targeting these biomarkers. Preliminary evidence has implicated brain circuitry that may mediate the reduction of suicide risk by treatments such as lithium and ketamine [182–184]. For example, ketamine-induced reductions in SI were associated with increases in baseline rCMRglu in a cluster including the cerebellum and occipital cortex [150]. In addition, in 57 adults with BD with and without a history of attempt, lowest DLPFC, OFC, ACC, superior temporal cortex, parietal and occipital cortex volumes were observed in SAs off-lithium, followed by SAs and nonattempters on lithium, with the largest volumes in people with nonattempting BD on lithium [34]. However, the study of other pharmacological and nonpharmacological treatments that can directly (e.g., deep brain stimulation) or indirectly target the involved circuitry is needed.

Although adolescents have been less studied than adults, some findings have been similar. For instance, alterations in structure of the VMPFC, VLPFC, DLPFC, DMPFC, ACC, lateral temporal regions, and parahippocampal gyrus were observed in both adolescents and adults with SAs. Furthermore, lower connectivity in regions related to the default mode network during rest have been consistently reported across adolescents, young adults, and adults with SI and/or SAs. Greater DLPFC activation in response to angry faces was also observed in both adolescents and adults with STBs. The highly limited number of studies in adolescents and a lack of different life stages in single studies prevents drawing conclusions about the overlap in functional brain alterations across different stages of life and brain maturation. Thus, there is some converging evidence across adolescent and adult samples, however, not all adult findings have been observed in adolescents (see Supplementary Tables 1–3 for a complete overview). This may at least in part be due to the continued maturation of involved brain systems so that not all features may be expressed until adulthood [185, 186].

Most studies only included a single disorder, impeding conclusions around shared vs. unique neural substrates of STBs across different mental disorders. Different studies employed different inclusion and exclusion criteria, imaging methods, and STB assessments. Nonetheless, gray matter alterations in the VLPFC, DLPFC, ACC, and insula have been consistently reported across the diagnostic categories reviewed (i.e., MDD, BPD, BD, and SZ), while lateral temporal alterations were more uniquely observed in psychotic disorders and BPD. Reduced white matter integrity in VPFC regions was reported in relation to STBs in both BD and MDD and lower FA in the corpus callosum was associated with a higher number of attempts across BD, MDD, and BPD. Functional brain alterations in relation to emotion processing and regulation was investigated only in MDD, BD, and BPD, with consistent findings of increased VLPFC activation in response to negative emotional stimuli across MDD and BPD, while the only study of adolescents with BD focused on amygdala-PFC connectivity [35]. Higher DLPFC activation during cognitive control tasks, in the absence of task performance differences, was also consistently reported across MDD, BD, SZ, and PTSD. In contrast, higher vs. lower IFG activation during cognitive control was observed in mood disorders vs SZ, respectively. Alterations in dACC connectivity with default mode, affective and reward network related regions in relation to SI, and alterations in dACC connectivity with DLPFC in relation to SAs during cognitive control, have been observed across MDD, BD, and SZ. Other functional domains such as reward processing, decision-making, social exclusion, and self-referential processing, as well as functioning at rest, have only been investigated in a single disorder, i.e., depression. In one of the few studies to assess SAs across MDD and BD (published subsequent to our literature search), VPFC gray matter volume reductions and uncinate fasciculus FA reductions were common to both disorders, suggesting that they may be important in risk for attempts across mood disorders [187]. Preliminary findings of that study also indicated there may be differences in involved regions in SAs between the disorders, with greater uncinate involvement in attempters in BD and dorsal frontal white matter in MDD. These findings suggest both transdiagnostic and unique gray and white matter targets for suicide prevention across mental disorders.

The examination of sex differences in STBs is a major gap in the neuroimaging literature and a critical area for study. There are well-established sex-dependent features known in STBs [188], such as a higher rate of attempts in females and a higher lethality of attempts in males [189–191]. Moreover, increased death rates by suicide between 1999 and 2017 as reported by the CDC showed the rate of increase was substantially higher for females than males (53% and 26%, respectively). Female suicide rate increases were particularly high in youth/early adulthood (ages 10–24 years) and middle age (45–64 years), when females have their highest risk, while the highest risk for males is age 75 years and older [192]. Of the studies in this review, 11 (8.4%) had exclusively female and 15 (11.5%) exclusively male samples. Authors of six studies (4.6%) commented on this homogeneity as a limitation, and 14 (10.7%) provided a rationale for single-sex studies. These included the need to avoid potential confounds of previously reported sex-based effects, e.g., previous reports of sex differences in cortical responses with similar fMRI tasks (*n* = 5) and corpus callosum structure (*n* = 2); high male representation in the group under investigation, such as military veterans (*n* = 3); female-skewed prevalence of BPD (*n* = 1); males and females imaged on different scanners (*n* = 1); small number of male SAs (*n* = 1); and an attempt to replicate previous work (*n* = 1). Ninety studies (68.7%) included sex as a covariate, controlling for potential differences. A small percentage of studies (13.8%) included sex as a variable to assess potential interactions with STBs. Five studies (3.8%) reported sex-related findings, though no relationship with STBs [36, 146, 193–196] and one study (0.8%) reported more females than males studied were SAs but reported no neuroimaging-related findings [197]. Chase et al. [198], in a study that controlled for sex, noted that one participant identified as transmale. Careful consideration is warranted in how sex and gender are evaluated and categorized for analyses, including the importance of allowing subjects to identify by gender and that this self-identification of gender is considered in all studies. This is particularly important because transgender and sexual-minority individuals are at increased risk for STBs and death by suicide [199, 200]. Collectively, these STB neuroimaging research data highlight the urgent need for future work on sex and gender.

Integration of neuroimaging research across differential mechanistic levels including genetic, molecular, social, and environmental risk factors will be crucial to the elucidation of a holistic view of STB pathophysiology and mechanisms associated with vulnerability and resilience and thus tailored intervention development. Sophisticated analytic methods, such as machine learning techniques [59], can be utilized to allow imaging risk markers to be identified at the level of the individual instead of at a group level, a key ingredient for clinically viable biomarkers [201] and precision medicine. In addition, the use of high-resolution ultrahigh field strength MRI methods and more specific functional neuroimaging tasks may further enhance ability to parse roles of specific brain regions, such as PFC subregions [202]. Molecular imaging has produced important leads, such as in serotonergic and inflammation mechanisms (Supplementary Table 2), consistent with postmortem, genetic, and peripheral biomarker studies implicating an important role of the serotonergic system and inflammatory mechanisms in STBs [203, 204]. Imaging of molecular mechanisms other than serotonin remains scarce, with for example only four magnetic resonance spectroscopy (^1^H-MRS) studies examining glutamatergic and GABA-ergic mechanisms published to date (Supplementary Table 2). Given postmortem findings of altered glutamatergic and GABA-ergic gene expression and receptor availability in suicide victims mainly in the PFC, ACC, and hippocampus [205–207], future ^1^H-MRS should clarify the role of these neurotransmitters in STBs in vivo. In addition, a generation of new methods to identify other currently implicated and novel molecular mechanisms is needed. For example, a link between oxytocin and SI was recently suggested [208].

Future studies should also further investigate the more elusive subjective aspects of suicide risk, such as SI, as well as implicated psychological constructs such as hopelessness, rumination, and anhedonia [209–211], which may be relevant across diagnoses. Similarly, social experiences such as childhood maltreatment and peer bullying form a strong prelude to STB in later life [208, 212, 213], and impact on the neural structures implicated in STB (e.g., DMPFC structure and function [214, 215]). Therefore, adverse experiences should be taken into account in future studies on the neurobiology of STB.

Links identified between neuroimaging measures and behaviors outside of the scanner could facilitate the development of less invasive and more easily and widely disseminated risk detection methods. Furthermore, investigations of larger samples can be facilitated by international collaborations that pool existing data across many different samples, such as the MQ HOPES (https://www.mqmentalhealth.org/research/profiles/overcome-and-predict-the-emergence-of-suicide) and ENIGMA STB (http://enigma.ini.usc.edu/ongoing/enigma-stb/) consortia. These initiatives represent cost-effective ways to substantially increase statistical power, which could provide more robust and reliable findings [216], and the ability to study age, gender, and sex effects, as well as unique and shared mechanisms associated with STBs across different mental disorders. Furthermore, large combined datasets will provide a unique opportunity to identify and test reproducibility of different pathways to suicide that may differ across many parameters including a range from the impulsive to the highly planned. Such efforts will need to take into account the variety of assessment methods used for STBs across studies. One way to address this important challenge is through the examination of the presence vs. absence of suicidal behavior (attempts), and/or SI (with or without intent and/or a plan) across assessment types (see for example Renteria et al. [110]). Another approach could involve standardization of scores across different instruments by developing a common metric [217]. Standardization of STB assessment across future studies would significantly facilitate the sharing of data, and thereby advance our understanding of brain-based STB vulnerability.

## Conclusions

More than 2 decades of neuroimaging studies on STBs suggests a transdiagnostic model for STBs in which an extended VPFC system may be important in the excessive negative and blunted positive internal states that can stimulate SI and a DPFC/IFG system that may facilitate SA behaviors. Interactions between these systems are likely important in the transition from ideation to attempt, perhaps mediated by dACC and insula regions, but require further investigation. With the exponential growth of research on STBs, including the initiation of large global efforts, it is hopeful that suicide prevention will soon be more effectively targeted, reducing the tragic loss of life to suicide.

## Supplementary information

Supplementary Tables
