# Supplementary material for: Imaging suicidal thoughts and behaviors: a comprehensive review of 2 decades of neuroimaging studies
Source: Mol Psychiatry. 2019 Dec 2;25(2):408–27. doi: 10.1038/s41380-019-0587-x (PMC6974434; doi:10.1038/s41380-019-0587-x)
Supplement: Supplementary file 1 — Supplementary Tables [file 41380_2019_587_MOESM1_ESM.pdf]

Supplementary Table S1. Findings from Structural Imaging Studies of Suicidal Thoughts and Behaviors

Authors, year*	Mental disorder	SA group/ SI group	Groups w/o SA and/or SI	Female n (%)**	Age	Methods	Findings***
**STRUCTURAL MAGNETIC RESONANCE IMAGING STUDIES OF GRAY AND WHITE MATTER**							
Suicide attempt studies							
Goodman et al 2011[1]	BPD + MDD	13 SA	13 HC	20 (77)	SA: 15.8 (1.1), HC: 16.2 (0.8)	ROI volume: OFC, ACC, DMPFC, DLPFC	Number of SA associated with ↓ ACC (BA24) volume (combined WM and GM), and ↑WM (not GM) in posterior cingulate (BA 23), however, similar association with BPD symptom severity for both findings
Fradkin et al 2017[2]	MDD	29 SA	29 HC	46 (79)	SA: 17.6 (1.82), HC:16.9 (1.72)	WB cortical thickness and surface area	In HC: motor impulsivity associated with ↑ RMPFC/RLPFC thickness. In SA: motor impulsivity associated with ↓ RMPFC/RLPFC thickness. In SA: non-planning impulsivity associated with ↑ paracentral lobule thickness. No findings medicated vs unmedicated
Cao et al 2016[3]	DD	35 SA	18 DC, 47 HC	66 (66)	SA: 20.63 (3.65), DC: 21.39 (3.05), HC: 20.53 (1.84)	WB VBM GMV and WMV (SPM)	SA + SI vs DC + SI: No differences between groups
Johnston et al 2017[4]	BD	26 SA	42 DC, 45 HC	43 (63)	SA: 20.5(3.0), DC:20.6 (3.2), HC:20.8(3.3)	WB, VBM GMV (SPM)	SA +SI vs DC: ↓ GMV in right medial/lateral OFC (BA11/47), hippocampus, bilateral cerebellum
Pan et al 2015[5]	MDD	28 SA	31 DC, 41 HC	ND	SA: 16.0 (1.27), DC: 16.06 (1.47), HC: 14.48 (1.84)	WB GMV, WMV and cortical thickness	SA + SI vs DC: ↓ caudal middle frontal gyrus (BA8) volume, temporal pole (BA38) thickness, parahippocampal gyrus (BA34) volume
Peng et al 2014[6]	MDD	20 SA	18 DC, 28 HC	38 (58)	SA: 27.75 (7.21), DC: 31.06 (7.39), HC: 28.61 (5.45)	WB VBM GMV (SPM)	SA vs DC (SI not reported): ↓ left PCC, which correlated negatively with dysfunctional attitudes
Gosnell et al 2016[7]	MDD, BD, AA, ANX	20 SA	20 DC, 20 HC	28 (47)	SA: 28.9 (9.98), DC: 29.25 (11.1), HC: 28.9 (10.0)	ROI volume: thalamus, insula, basal ganglia, hippocampus, amygdala, corpus callosum, cortical lobes.	SA vs DC: ↓ right precentral gyrus, right IFG, right caudal middle frontal gyrus (DPFC), left precentral lobule and total temporal cortex. No association between SI (regardless of history of SA) and any of the ROIs
Soloff et al 2012[8]	BPD	44 SA	24 DC, 52 HC	76 (63)	SA: 29.6 (8.0), DC: 25.9 (5.7), HC: 25.9 (7.2)	ROI VBM GMV (SPM), ROIs: IFG, OFC, ACC, middle and superior temporal cortex, insula, hippocampus, parahippocampus, fusiform gyrus, lingual gyrus and amygdala	SA vs DC: ↓ insula and larger lingual and middle and superior temporal gyri. In SA: HL associated with ↓ lateral OFC, middle and superior temporal gyri, insula, fusiform gyrus, lingual gyrus and parahippocampal gyrus
Monkul et al 2007[9]	MDD	7 SA	10 DC, 17 HC	34 (100)	SA: 31.4 (13.9), DC: 36.5 (7.5), HC: 31.3 (8.3)	ROI manual shape tracing, ROIs: OFC, ACC, PCC, amygdala and hippocampus. DLPFC, subgenual PFC, thalamus, temporal lobe, caudate and lateral ventricles in exploratory analyses	SA vs DC: ↑ right amygdala

Authors, year*	Mental disorder	SA group/ SI group	Groups w/o SA and/or SI	Female n (%)**	Age	Methods	Findings***
Besteher et al 2016[10]	SCZ	14 SA	23 DC, 50 HC	46 (53)	SA: 34.4 (12.1), DC: 28.8 (9.7), HC: 29.5 (7.9)	WB cortical thickness and mean curvature	SA vs DC: SA ↓ in right DLPFC and superior and middle temporal gyri, temporopolar cortex, and insula
Giakoumatos et al 2014[11]	SCZ, SZA or BD-P	148 SA (97 HL, 51 LL)	341 DC, 262 HC	387 (52)	SA-HL: 35.6 (11.7), SA-LL: 36.9 (12.2), DC: 35.9 (13.3), HC: 38.1 (12.5)	WB GMV, cortical surface area and thickness	SA vs DC: ↓ GMV in bilateral superior and middle frontal gyri (DLPFC), and inferior and superior temporal gyri, left superior parietal and supramarginal cortex, and right insula and thalamus. High (vs. low) lethality: ↓ GMV in left lingual area and right cuneus across all attempters; left dorsal ACC (BA32), left inferior parietal cortex, left inferior temporal gyrus, and right middle temporal gyrus in BD-P; left lingual gyrus, bilateral pericalcarine, right cuneus and right lateral occipital cortex in SZ; left middle frontal gyrus (DLPFC) in SZA.
Rüsch et al 2008[12]	SCZ	10 SA	45 DC, 55 HC	42 (38)	SA: 30.3 (6.5): DC 37.3 (11.6)	WB VBM GMV and WMV (SPM)	SA vs DC: ↑ WMV in bilateral posterior lateral OFC and IFG. No GMV differences
Matsuo et al 2010[13]	BD	10 SA	10 DC, 27 HC	47 (100)	SA: 36.2 (10.1), DC:44.2 (12.5), HC: 36.9 (13.8)	ROI volume, manual shape tracing of CC genu, anterior body, posterior body, isthmus and splenium	SA vs DC: no significant differences. In SA: impulsivity associated with ↓ anterior corpus callosum genu
Lijffijt et al 2014[14]	BD	51 SA	42 DC, 45 HC	138 (100)	SA: 36.6 (10.7), DC: 41.1 (11.3)	ROI volume, ROIs: superior frontal gyrus, rostral and caudal middle frontal gyrus, frontal pole, IFG, medial and lateral OFC, rostral and caudal ACC	SA vs DC: no differences. Lower PFC GMV only in SA with previous hospitalization
Soloff et al 2014[15]	BPD	51 SA (16 HL, 35 LL)		41 (80)	SA-HL: 36.1 (9.2): SA-LL: 27.4 (5.9).	ROI volume VBM of GMV (SPM), ROIs: OFC, ACC, middle and superior temp gyrus, insula, (para-)hippocampus, lingual gyrus, amygdala	HL vs LL SA: ↓ GMV in bilateral middle and superior temporal gyri, left lingual gyrus, bilateral lateral OFC, right insula, bilateral fusiform gyrus, right parahippocampus, left ventral and dorsal ACC and left hippocampus. In HL SA: aggression associated with ↑ ventral and dorsal ACC, lateral OFC, middle and superior temporal gyri and right insula, and impulsivity associated with ↑ right middle and superior temporal gyri and ↓ insula. In LL SA: impulsivity associated with ↓ right middle and superior temporal gyri, bilateral insula, bilateral lingual gyrus, ventral ACC, fusiform gyrus, lateral OFC, hippocampus and amygdala
Aguilar et al 2008[16]	SCZ	13 SA	24 DC	0 (0)	SA: 37.1 (11.0), DC: 42.7 (10.2)	WB VBM GMV (SPM)	SA vs DC: ↓ medial OFC and superior temporal gyrus
Ding et al 2015[17]	Past MDD/BD	67 SA	82 DC, 82 HC	98 (42)	SA: 39.2 (10.6), DC: 39.4 (9.7),HC: 37.8 (8.1)	WB and ROI VBM GMV (SPM) and cortical thickness and surface area, ROIs: OFC, VLPFC, VMPFC (including ACC), and DLPFC	SA vs DC: ↓ OFC (BA47, lateral part of BA11) in exploratory WB analysis. In SA: lethality last SA associated with ↓ right DPFC BA8/9/46), OFC, left VLPFC (BA44/45). Number SA associated with ↓ right DPFC and left OFC. No associations with SI.

Authors, year*	Mental disorder	SA group/ SI group	Groups w/o SA and/or SI	Female n (%)**	Age	Methods	Findings***
Gifuni et al 2017[18]	Past MDD/BD	61 SA	75 DC, 73 HC	120 (47)	SA: 38.3 (10.7), DC: 38.4 (9.1), HC: 39.2 (7.0)	ROI volume SBM, ROI: corpus callosum	SA vs DC: no differences. No correlation corpus callosum volume and SI, age at first SA and number of SA
Gifuni et al 2016[19]	Past DD/BD	73 SA	89 DC, 91 HC	120 (47)	SA: 39.2 (10.6), DC: 39.4 (9.5), HC: 38.3 (8.2)	ROI volume, ROIs: amygdala, hippocampus, caudate, globus pallidus, putamen, nucleus accumbens, ventral diencephalon and thalamus	SA vs DC: no differences. In SA: lethality SA associated with ↓ left and right nucleus accumbens
Harenski et al 2017[20]	SCZ, SZA, BD-P, or MDD-P	18 SA	18 DC, 59 HC, 26 HC	0 (0)	SA: 38.9 (11.73), DC: 40.2 (10.23), HC: 32.5 (11.16), CHC: 33.0 (9.49)	WB and ROI VBM GMV (SPM), ROIs: posterior superior temporal cortex, temporal poles and medial PFC	SA vs DC: ↓ left and right temporal pole
Nery-Fernandes et al 2012[21]	BD	19 SA	21 DC, 22 HC	41 (66)	SA: 39.8 (11.4), DC: 42.0 (8.6), HC: 37.7 (13.5)	ROI volume VBM GMV (SPM), ROI: corpus callosum	SA vs DC: no differences
Vang et al 2010[22]	MDD, AD	7 SA	6 HC	ND	SA: 40 (11.83), matched HC, no details	ROI volume, ROIs: subcortical structures	SA vs HC: ↓ globus pallidus and caudate, and correlated with 5-HTT binding. In SA: non-impulsive temperament associated with ↓ globus pallidus GMV
Baldaçara et al 2011[23]	BD	20 SA	20 DC, 22 HC	41 (66)	SA: 39.94 (11.2), DC: 41.9 (8.9), HC: 37.7 (13.6)	ROI volume VBM GMV and WMV (SPM), ROIs: cerebellum	SA vs DC: no differences total brain volume or cerebellar volume
Wagner et al 2011[24]	MDD	10 with SA and/or first-degree relative with SA	15 DC, 30 HC	50 (83)	SA: 41.0 (12.5): DC: 34.1 (10.5), HC: 35.1 (10.4).	WB VBM GMV(SPM)	SA vs DC: ↓ rostral ACC (BA24) and right caudate
Wagner et al 2012[25]	MDD	10 with SA and/or first-degree relative with SA	15 DC, 30 HC	50 (83)	SA: 41.0 (12.5), DC: 34.1 (10.5), HC: 35.1 (10.4)	WB cortical thickness	SA vs DC: ↓ VLPFC (BA47), DLPFC (BA46) and dorsal ACC (BA32). Patients with own versus relative with SA: no differences in dorsal ACC, VLPFC, DLPFC
Duarte et al 2017[26]	BD	20 SA	19 DC, 20 HC	34 (57)	SA: 41.10 (12.64): DC: 42.26 (11.70), HC: 37.40 (10.20)	WB and ROI volume VBM GMV (SPM), ROIs: OFC, DLPFC (including IFG), ACC, amygdala, hippocampus, thalamus and insula	SA vs DC: ↑ right rostral ACC (BA24). In SA: HL SA was associated with ↑ insula, LL SA was associated with ↓ OFC (BA47)

Authors, year*	Mental disorder	SA group/ SI group	Groups w/o SA and/or SI	Female n (%)**	Age	Methods	Findings***
Benedetti et al 2014[27]	BD	32 SA	104 DC	93 (68)	SA (5-HTTLPR l/l): 41.4 (10.7), SA (5-HTTLPRs): 46.4 (12.8), DC (5-HTTLPR l/l): 48.5 (10.4), DC (5-HTTLPRs): 46.8 (12.6)	WB GMV (SPM)	SA vs DC: no differences
Lee et al 2016[28]	MDD	19 SA	19 DC, 20 HC	41 (73)	SA: 42.0 (10.8), DC: 41.1 (15.2)	ROI volume GMV (SPM), ROIs not specified	SA vs DC: ↓ right cerebellum and left angular gyrus
Spoletini et al 2011[29]	SCZ	14 SA	36 DC, 50 HC	35 (39)	SA: 42.9 (11.3), DC: 39.8 (11.4): HC: 40.0 (16.6)	ROI volume GMV (FSL), ROIs: lateral ventricles, thalamus, caudate, putamen, pallidum, hippocampus, amygdala, nucleus accumbens	SA vs DC: ↑ right amygdala
Benedetti et al 2011[30]	BD	19 SA (with/without Lithium)	38 DC (with/without Lithium)	38 (67)	SA-L-: 43.6 (10.4), SA-L+: 45.6 (11.3), DC-L-: 45.9 (10.5), DC-L+: 46.2 (13.3)	WB VBM GMV (SPM)	SA vs DC: ↓ DPFC (BA6/8/9), RLPFC (BA10), OFC (BA11/47), dorsal ACC (BA32), parietal and occipital cortex and ↑ in bilateral superior temporal gyrus. SA with lithium vs without: ↑ DPFC (BA6/8), OFC (BA11/47), ACC (BA24/32), parietal and occipital cortex and ↓ in bilateral superior temporal gyrus
Colle et al 2015[31]	MDD	24 SA	39 DC	39 (62)	SA: 44.2 (11.9), DC: 47.7 (12.6)	ROI volume GMV (SACHA, automatic segmentation), ROI: hippocampus.	SA vs DC: ↓ hippocampus. No difference between SA in last month versus SA >1 month ago
Dombrovski et al 2012[32]	MDD	13 SA	20 DC, 19 HC	30 (58)	SA: 66.0 (6.4), DC: 67.7 (7.0), HC: 70.5 (7.5)	ROI voxel count basal ganglia (caudate, putamen, pallidum)	SA vs DC: ↓ putamen, associative and ventral striatum voxel count. In SA: delay discounting associated with ↓ putamen voxel count
Cyprien et al 2011[33]	MDD, ANX, BD	21 SA or SI	234 DC, 180 HC	222 (51)	SA: 72.2 (4.3), DC: 71.0 (3.8), HC: 71.0 (3.8)	ROI volume manual shape tracing, ROI: corpus callosum	SA vs DC: ↓ posterior third of corpus callosum
Hwang et al 2010[34]	MDD	27 SA	43 DC, 26 HC	0 (0)	SA:79.1 (5.6), DC:79.6 (5.1), HC: 79.5 (4.3)	WB VBM GMV and WMV (SPM)	SA vs DC: ↓ GMV in DPFC (BA6/8/9/46), precentral gyrus, postcentral gyrus, superior parietal lobe, inferior parietal lobe, cuneus, superior temporal gyrus, insula, cerebellum, midbrain, ↓ WMV in DPFC (BA6/8/9/46), precentral and postcentral gyrus, inferior parietal lobe, precuneus, occipital lobe, external capsule, cerebellum
Lopez-Larson et al 2013[35]	TBI	19 SA	40 DC, 15 HC	0 (0)	18-55	ROI volume, ROI: thalamus	SA vs DC: ↑ right thalamus
Jia et al 2010[36]	MDD	16 SA	36 DC, 52 HC	55 (53)	SA: 34.2 (13.7), DC: 34.7 (12.5), HC: 37.1 (16.0)	WB VBM GMV and WMV (SPM)	SA vs DC: no differences

Authors, year*	Mental disorder	SA group/ SI group	Groups w/o SA and/or SI	Female n (%)**	Age	Methods	Findings***
Kim et al 2015[37]	PD	12 SA	25 DC	23 (64)	16-60	WB VBM GMV and WMV (SPM)	SA vs DC: no differences
Rentería et al 2017[38]	MDD	153 SA or SI+plan, 298 SI-plan	650 DC, 1996 HC	ND	SA+SI: 21.10-53.8 (across 7 samples), DC: 22.9-54.8, HC: 22.9-55.4.	ROI volume, ROIs: nucleus accumbens, amygdala, caudate, hippocampus, pallidum, putamen, thalamus, ICV	SA+SI with plan vs DC: no differences. SI vs DC: no differences
Suicidal ideation studies							
Thomas et al 2004[39]	PTSD + Childhood maltreatment	47 SI, 17 SA	14 DC, 121 HC	89 (49)	DC: 11.71 (2.6): HC: 11.74 (2.5)	ROI volume manual shape tracing (IMAGE), ROI: pituitary	SI (with SA) vs DC: ↑ pituitary
Taylor et al 2015[40]	MDD	21 SI, including 10 with past SA	53 DC, 91 HC	108 (65)	SI: 33.5 (9.1), DC:37.5 (8.9), HC: 29.9 (9.1)	WB and ROI volume GMV and cortical thickness, ROIs: OFC, cingulate cortex, insula, amygdala, parahippocampus, thalamus, basal ganglia	SI+SA vs DC: ↓ cortical thickness of left insula, left caudal middle frontal gyrus (DLPFC), left superior parietal cortex, left superior temporal gyrus. No GMV differences
Caplan et al 2010[41]	Epilepsy & MDD, ANX, ADHD	11 SI (No past SA)	40 DC	28 (55)	SI: 11.04 (2.06), DC:9.43 (2.07)	ROI volume manual shape tracing, ROIs: middle frontal gyrus, superior frontal gyrus, OFC, temporal lobe	SI vs DC: ↓ right orbital frontal gyrus WMV and ↑ left temporal lobe GMV
MAGNETIC RESONANCE IMAGING STUDIES OF WHITE MATTER HYPERINTENSITIES							
Suicide attempt studies							
Ehrlich et al 2003[42]	MDD, BD, PsD, conduct/ADHD	43 SA	110 DC	39 (26)	Entire sample: 14.6 (3.4). No further details provided.	WMH (Modified version of Coffey scale)	SA vs DC: ↑ DWMHs in parietal lobes. All SA subjects had lesions in right posterior parietal lobe
Ehrlich et al 2004[43]	MDD, BD, PsD, conduct/ADHD	43 SA	110 DC	41 (27)	Entire sample: 14.6 (3.4). No further details provided.	WMH (Modified version of Coffey scale)	↑ WMH associated with past SA, driven by PVH. SI not associated with WMH
Ehrlich et al 2005[44]	MDD	62 SA	40 DC	68 (67)	Entire sample: 26.7 (5.5). No further details provided.	WMH (Modified version of Fazekas scale)	SA vs DC: ↑ PVH, not DWMH. No association with SI
Pompili et al 2007[45]	MDD, BD	29 SA	26 DC	41 (63)	SA: 42.2 (13.5), DC: 44.6 (14.0)	WMH (Modified version of Fazekas scale)	SA vs DC: ↑ WMH. SI was not associated with WMH
Pompili et al 2008[46]	MDD, BD	44 SA	55 DC	57 (58)	SA: 45.57 (16.10), DC: 47327(14.54)	WMH (Modified version of Fazekas scale)	SA vs DC: ↑ PVH, no difference DWMH

Authors, year*	Mental disorder	SA group/ SI group	Groups w/o SA and/or SI	Female n (%)**	Age	Methods	Findings***
Ahearn et al 2001[47]	MDD	20 SA	20 DC	17 (85)	SA: 66.0 (5.8), DC: 66.4 (5.7)	WMH (Coffey and Boyko scales)	SA vs DC: ↑ subcortical GM hyperintensities, and trend towards more PVH
Sachs-Ericsson et al 2014[48]	MDD	23 SA	223 DC	149 (67)	SA: 66.74 (6.6), DC: 69.8 (7.5)	WMH (Duke Neuropsychiatric Imaging Research Laboratory modified version of MrX software)	SA vs DC: ↑ WM lesions in the left hemisphere. ↑ increase over time in bilateral WMH in SA, which was predicted by the number of depressive episodes
DIFFUSION TENSOR IMAGING STUDIES							
Suicide attempt studies							
Johnston et al 2017[4]	BD	26 SA	42 DC, 45 HC	43 (63)	14-25	WB FA maps (SPM)	SA vs DC: ↓ FA in left uncinate fasciculus, right uncinate fasciculus and right cerebellum
Lischke et al 2017[49]	BPD	13 SA	8 DC, 20 HC	41 (100)	18-45	Tractography based FA and MD with seeds in genu, splenium and body of corpus callosum	SA vs DC: no differences. Number of attempts associated with ↓ FA and MD in splenium and FA in the genu
Lee et al 2016[50]	SCZ	15 SA	41 DC	41 (73)	18-60	WB FA, MD, AD and RD maps (FSL)	SA vs DC: ↑ FA in left corona radiata (anterior, superior, posterior), superior longitudinal fasciculus, posterior limb and retrolenticular part of internal capsule, external capsule, posterior thalamic radiation, sagittal stratum (including inferior longitudinal fasciculus and inferior fronto-occipital fasciculus), ↓ AD in retrolenticular part of internal capsule, posterior thalamic radiation and sagittal stratum. No differences in MD and RD
Kim et al 2015[37]	PD	12 SA	25 DC	23 (64)	16-60	FA, MD, AD and RD maps (FSL), Tracts: corona radiata, inferior longitudinal fasciculus, inferiorfronto-occipital fasciculus, superior longitudinal fasciculus, posterior thalamic radiation, internal capsule, splenium of corpus callosum	SA vs DC: ↑ FA in posterior and superior corona radiata, sagittal stratum (including inferior longitudinal fasciculus and inferior fronto-occipital fasciculus), superior longitudinal fasciculus, posterior thalamic radiation, retrolenticular part of internal capsule and splenium of the corpus callosum. No differences in MD, RD and AD. In SA: positive correlation between suicidal ideation and FA of the right retrolenticular part of internal capsule and right and left posterior thalamic radiation. In DC: positive correlation between suicidal ideation and FA of splenium, right retrolenticular part of internal capsule and left posterior thalamic radiation
Mahon et al 2012[51]	BD	14 SA	15 DC, 15 HC	18 (41)	SA: 33.3 (14.1), DC: 36.5 (12.3), HC: 33.7 (12.6)	WB FA maps	SA vs DC: ↓ FA in white matter tract in medial VPFC. In SA: medial VPFC FA negatively correlated with motor impulsivity
Olvet et al 2014[52]	MDD	13 SA	39 DC, 46 HC	52 (53)	18-65	ROI FA and ADC maps (FSL), ROIs: mOFC, DMPFC, rACC, dACC	SA vs DC: ↓ FA in DMPFC. No difference in AD

Authors, year*	Mental disorder	SA group/ SI group	Groups w/o SA and/or SI	Female n (%)**	Age	Methods	Findings***
Jia et al 2010[36]	MDD	16 SA	36 DC, 52 HC	55 (53)	SA: 34.2 (13.7), DC: 34.7 (12.5), HC: 37.1 (16.0)	WB and ROI analysis of FA, MD and RD maps (DTIstudio), ROIs: bilateral lentiform nucleus, bilateral hippocampus, and bilateral thalamus	SA vs DC: ↓ FA and AD in the left anterior limb of internal capsule and ↓ FA and ↑ RD in right lentiform nucleus
Jia et al 2014[53]	MDD	23 SA	40 DC, 46 HC	59 (54)	SA: 36.3 (14.5, DC: 34.0 (14.5), HC: 33.3 (11.4)	Tractography based FA with seed in left anterior limb of the internal capsule	SA vs DC: ↓ percentage of projecting fibers connecting the anterior limb of the internal capsule to the left OFC and left thalamus
Lopez-Larson et al 2013[35]	TBI	19 SA	40 DC, 15 HC	0 (0)	18-55	ROI FA maps (FSL), ROI: anterior thalamic radiation	SA vs DC: ↑ FA in bilateral anterior thalamic radiation. Positive correlation impulsivity and FA in right anterior thalamic radiation
Cyprien et al 2016[54]	BD, MDD	45 SA	46 DC, 30 HC	121 (100)	18-50	ROI FA, MD, RD, AD maps (FSL), ROIs: genu, body and splenium of corpus callosum	SA vs HC: no differences that survived multiple comparison correction. Number of attempts associated with ↓ FA in genu, body and splenium of corpus callosum. FA in splenium negatively correlated with suicidal intent
Bijttebier et al 2015[55]	past MDD	13 SA	15 DC, 17 HC	32 (72)	18-65	Tractography combined with network-based statistics	SA vs DC: ↓ structural connectivity in network including medial VPFC, temporal gyrus, precuneus, cuneus, parietal cortex, amygdala, hippocampus, occipital regions. Decreased connectivity between left olfactory cortex and left ACC
Suicidal ideation studies							
Myung et al 2016[56]	MDD	24 SI	25 DC, 31 HC	52 (65)	18-62	Tractography combined with network-based statistics and graph analysis	SA vs DC: ↓ structural connectivity in left hemisphere network including striatal regions, frontal regions (DLPFC, IFG, lateral OFC and RMPFC), lateral occipital and superior parietal regions. Betweenness centrality of left rostral middle frontal gyrus (DLPFC) positively correlated with suicidal ideation. Participation coefficient of left rostral middle frontal gyrus (DLPFC) positively correlated with impulsivity
Taylor et al 2015[40]	MDD	21 SI, including 10 with past SA	53 DC, 91 HC	108 (65)	20-50	ROI FA and MD maps (FSL), ROIs: internal capsule, thalamic radiation, cingulum bundle, corpus callosum, uncinate fasciculus	SA vs DC: ↑ RD and ↓ FA in the corona radiata, the hippocampal region of the cingulum and the anterior thalamic radiation

Authors, year*	Mental disorder	SA group/ SI group	Groups w/o SA and/or SI	Female n (%)**	Age	Methods	Findings***

Symbols & Abbreviations: *Full citations can be found in the reference list below; **Percentages are rounded to the nearest whole number; ***Results are reported for SA or SI in comparison with diagnostic controls. If no diagnostic controls were included in the study, results based on SA or SI compared to healthy controls are reported.

AA: alcohol abuse; **ACC**: anterior cingulate cortex; **AD**: adjustment disorder; **ADHD**: attention deficit hyperactivity disorder; **ANX**: anxiety disorder; **BA:** Broadman's Area; **BD**: bipolar disorder; **BD-P**: bipolar disorder with psychotic symptoms; **BPD**: borderline personality disorder; **DC**: diagnostic controls; **DD**: depressive disorder; **DLPFC**: dorsolateral prefrontal cortex; **DMPFC**: dorsomedial prefrontal cortex; **DWMH**: deep white matter hyperintensities; **FA**: fractional anisotropy; **GM:** grey matter; **GMV**: grey matter volume; **HC**: healthy controls; **HL**: high lethality; **IFG**: inferior frontal gyrus; **LL**: low lethality; **MD**: mood disorder; **MDD**: major depressive disorder; **MDD-P**: major depressive disorder with psychotic features; **ND:** not detailed; **OFC**: orbitofrontal cortex; **PCC**: posterior cingulate cortex; **PD**: personality disorder; **PFC**: prefrontal cortex; **PsD**: psychosis; **PVH**: periventricular hyperintensities; **RLPFC**: rostrolateral prefrontal cortex; **RMPFC**: rostromedial prefrontal cortex; **ROI**: region of interest; **SA**: suicide attempt; **SBM**: surface based morphometry; **SCZ**: schizophrenia; **SI**: suicidal ideation; **SUD**: substance use disorder; **SZA**: schizoaffective disorder; **SPM**: Statistical Parametric Mapping toolbox; **TBI**: traumatic brain injury; **VBM**: voxel based morphometry; **VLPFC**: ventrolateral prefrontal cortex; **VMPFC**: ventromedial prefrontal cortex; **WB**: whole brain; **WM:** white matter; **WMH**: white matter hyperintensities; **WMV**: white matter volume; **w/o:** without; **5-HTTLPR l/l**: serotonin transporter long/long genotype; **5-HTTLPRs**: serotonin transporter s allele carriers genotype.

Supplementary Table S2. Findings from Molecular Imaging Studies of Suicidal Thoughts and Behaviors

Authors, year*	Mental disorder	SA group/ SI group	Groups w/o SA and/or SI	Female n (%)**	Age	Methods	Findings***
**SINGLE PHOTON EMISSION TOMOGRAPHY STUDIES**							
Suicide attempt studies							
Audenaert et al 2001[57]	MDD, AD, PsD	9 SA	12 HC	8 (38)	19-48	123I-5-I-R91150 for 5-HT2a receptors in PFC	SA vs HC: ↓ binding potential of 5-HT2a receptors in PFC
Audenaert et al 2002[58]	MDD	20 SA	20 HC	24 (60)	19-50	99mTc-Ethyl Cystine Dimer rCBF SPECT during letter and category fluency	SA vs HC: ↓ perfusion in IFG, ACC, temporal gyrus, hypothalamus during verbal fluency task
van Heeringen et al 2003[59]	MDD, AD, PsD	9 SA	13 HC	ND	19-47	123I-5-I-R91150 for 5-HT2a receptors in PFC	SA vs HC: ↓ binding potential of 5-HT2a receptors in PFC
Amen et al 2009[60]	MDD	12 SA	12 DC, 12 HC	3 (8)	19-64	99mTc HMPAO SPECT to assess rCBF	SA vs DC: ↓ rCBF in subgenual ACC, ↑ rCBF in right insula, dorsal ACC
Willeumier et al 2011[61]	MD	21 SA	36 DC, 27 HC	5 (24)	15-66	99mTc HMPAO SPECT to assess rCBF	SA vs DC: ↓ rCBF in frontal, temporal and parietal regions
Fountoulakis et al 2004[62]	MDD	13 SA, 10 SI	33 DC	ND	21-60	99mTc HMPAO SPECT to assess rCBF	SA vs DC: no differences. SI vs no-SI: no differences
Henningsson et al 2009[63]	MDD, PD	9 SA	9 HC	ND	23-67	123I-β-CIT for 5-HTT binding potential, assessment of Val66Met polymorphisms	Within SA: carriers of the Val/Val genotype of Val66Met had ↑ 5HTT binding potential in the parietal cortex and in the occipital lobes
Bah et al 2008[64]	MDD, AD, PD	9 SA	9 HC	0 (0)	23-67	123I-β-CIT for 5-HTT binding potential, assessment of SLC6A4 polymorphisms	SA vs HC: no differences. In SA: presence of S-allele of 5-HTTLPR genotype associated with lower 5-HTT binding potential in frontal, parietal and occipital cortex
Lindström et al 2004[65]	MDD, AD, PD	12 SA	12 HC	4 (17)	23-67	123I-β-CIT methods to separate 5-HTT and DAT uptake	SA vs HC: no differences in 5-HTT or DAT binding. In SA: impulsivity associated with ↓ whole brain 5-HTT binding
Ryding et al 2006[66]	MDD, AD, ANX, PD	12 SA	12 HC	4 (17)	23-67	123I-β-CIT methods to separate 5-HTT and DAT uptake	SA vs HC: no differences in 5-HTT or DAT binding. In SA: impulsivity associated with ↓ 5-HTT binding potential in inferior/orbital frontal cortex, temporal regions, midbrain, thalamus, basal ganglia
Vang et al. 2010[22]	MDD, AD	7 SA	6 HC	3 (23)	SA: 40 (11.83), matched HC, no details	123I-β-CIT methods to separate 5-HTT and DAT uptake	SA vs HC: not reported. In SA: significant negative correlation between 5HTT binding and globus pallidus volume
Suicidal ideation studies							
Fountoulakis et al 2004[62]	MDD	13 SA, 10 SI	33 DC	ND	21-60	99mTc HMPAO SPECT to assess rCBF	SI vs DC: no differences. SI vs no-SI: no differences

Authors, year*	Mental disorder	SA group/ SI group	Groups w/o SA and/or SI	Female n (%)**	Age	Methods	Findings***
POSITRON EMISSION TOMOGRAPHY STUDIES							
Suicide attempt studies							
Soloff et al 2003[67]	BPD	13 SA	9 HC	22 (100)	18-49	[18F]FDG PET during rest	SA vs HC: ↓ rCMRglu in bilateral medial OFC
Yeh et al 2015[68]	MDD	5 SA	5 DC, 10 HC	0 (0)	20-25	4-[18F]-ADAM for SERT availability	SA vs DC: ↑ SERT binding potential in the midbrain, thalamus, striatum and PFC. Suicidal ideation associated with ↑ SERT binding potential in the same 4 regions
Soloff et al 2014[69]	BPD	21 SA	12 DC, 27 HC	32 (53)	BPD: 27.5 (7.2), HC: 28.8 (8.2)	[18F]altanserin for 5-HT2a receptor binding potential	SA vs DC: ↑ binding potential of 5-HT2a receptors in the occipital cortex in females only
Soloff et al 2007[70]	BPD	12 SA	2 DC, 11 HC	25 (100)	19-46	[18F]altanserin for 5-HT2a receptor binding potential	SA vs HC: ↑ binding potential of 5-HT2a receptors in the hippocampus, medial temporal cortex and occipital cortex. No associations with number of attempts
Cannon et al 2006[71]	BD	8 SA	10 DC, 37 HC	36 (65)	BD: 30 (9), HC: 32 (9)	[11C]DASB for 5-HTT binding potential	SA vs DC: ↓ 5-HTT binding in the midbrain and ↑ in the rostral ACC
Oquendo et al 2003[72]	MDD	25 SA (9 LL, 16 HL)		15 (60)	LL: 30.4 (8.7), HL: 42.9 (10.4)	[18F]FDG PET, fenfluramine vs. placebo challenge	HL vs LL SA: ↓ rCMRglu in ACC (BA24/32), IFG (BA44) and DPFC (BA6/8/9), more pronounced after fenfluramine challenge. Lower VMPFC rCMRglu associated with lower impulsivity, higher suicidal intent and higher lethality
Sullivan et al 2015[73]	MDD	29 SA	62 DC	59 (65)	18-65	[11C]WAY-100635 for 5-HT1a receptor binding potential	SA vs DC: no differences in 5-HT1a receptor binding. High vs Low lethality SA: ↑ 5-HT1a receptor binding potential in the raphe nuclei. Positive association 5-HT1a receptor binding potential in the raphe nuclei and suicidal intent. Positive association 5-HT1a receptor binding potential in the raphe nuclei and PFC and suicidal ideation
Miller et al 2016[74]	BD, MDD	11 SA	6 DC, 31 HC	29 (60)	21 - 61	[11C]DASB for 5-HTT binding potential	SA vs DC: no differences in 5-HTT binding
Yeh et al 2015[75]	MDD	8 SA	9 DC, 17 HC	18 (53)	20-65	4-[18F]-ADAM for SERT availability	SA vs DC: no differences in SERT binding in individual regions, but ↑ PFC/midbrain SERT binding ratio. Suicide intent positively associated with PFC/midbrain SERT binding ratio
Leyton et al 2006[76]	MD, PD, SUD (all HL)	10 SA	16 HC	7 (28)	SA: 37.7 (6.4), HC: 35.5 (12.0)	Alpha-11C-methyl-L-tryptophan trapping as index of 5-HT synthesis	SA vs HC: ↓ 5-HT synthesis in lateral and medial OFC extending into VMPFC, ↑ 5-HT synthesis in thalamus, paracentral lobule, occipital cortex and hippocampus. Negative correlation suicide intent and serotonin synthesis in lateral OFC and VMPFC
Parsey et al 2006[77]	MDD	9 SA	16 DC, 43 HC	49 (69)	MDD: 38.0 (13.4), HC: 38.8 (15.9)	[11C]McN 5652 for 5-HTT binding potential	SA vs DC: no differences in SERT binding

Authors, year*	Mental disorder	SA group/ SI group	Groups w/o SA and/or SI	Female n (%)**	Age	Methods	Findings***
Miller et al 2013[78]	MDD	15 SA	36 DC, 32 HC	41 (49)	SA: 38.5 (11.5), DC: 41.0 (10.5), HC: 32.6 (11.3)	[11C]DASB for 5-HTT binding potential	SA vs DC: ↓ 5-HTT binding potential in midbrain
Nye et al 2013[79]	MDD	11 SA	10 HC	8 (38)	SA: 38.5 (13.6), HC: 21.3 (2.4)	[11C]ZIENT for SERT binding potential	SA vs HC: ↓ 5-HTT in the midbrain/pons and putamen
Mann et al 2018[80]	MDD	8 SA	8 DC, 8 HC	ND	21-53	[11C]WAY-100635 for 5-HT1a receptor binding potential, [18F]altanserin for 5-HT2a receptor binding potential	SA vs DC: no difference in 5-HT1a and 5-HT2a receptor binding
Oquendo et al 2016[81]	MDD	51 past SA, 15 future SA	49 DC	61 (61)	18-65	[11C]WAY-100635 for 5-HT1a receptor binding potential, [11C]DASB for 5-HTT binding potential	Future attempt versus no future attempt: no differences in 5-HTT binding. Higher lethality of future attempts associated with ↑ 5-HT1a receptor binding potential in insula, DPFC, ACC and raphe nuclei. SI at follow up associated with ↑ 5-HT1a receptor binding in raphe nuclei, amygdala, hippocampus, parahippocampul gyrus, temporal lobe, ACC, DPFC, medial PFC, OFC, insula, occipital lobe, parietal lobe
Sublette et al 2013[82]	MDD, BD	13 SA	16 DC	19 (66)	SA: 36.0 (11.5), DC: 42.2 (13.0)	[18F]FDG PET, fenfluramine vs. placebo challenge	SA vs DC: ↓ rCMRglu in right DLPFC, more pronounced after fenfluramine challenge, and ↑ rCMRglu in VMPFC, not more pronounced after fenfluramine. SI negatively correlated with rCMRglu in DLPFC
Suicidal ideation studies							
Holmes et al 2018[83]	MDD	9 SI	5 DC, 13 HC	13 (48)	MDD: 31 (12), HC: 33 (11)	[11C](R)-PK11195 for TSPO availability index of neuroinflammation)	SI vs no-SI: ↑ TSPO availability in ACC and insula
Kolla et al 2016[84]	BPD	ND	28 DC, 14 HC	56 (100)	18-51	[11C]Harmine for MAO-A VT (index of MAO-A density)	Positive association MAO-A VT in PFC and ACC with SI, but also with depressive symptom scores
Van Heeringen et al 2017[85]	MDD	17 SI + plan, 11 SI	12 DC, 20 HC	38 (63)	SI+plans: 46.1 (10.9), SI: 42.6 (11.6), DC: 51.2 (6.5), HC: 43.8 (13.1)	[18F]FDG PET during rest	SI+plans vs SI: ↓ rCMRglu in RLPFC and inferior parietal lobe
Ballard et al 2015[86]	MDD	12 SI	8 DC	6 (30)	MDD: 48 (12)	[18F]FDG PET during rest, ketamine challenge	Baseline SI associated with ↑ rCMRglu in infralimbic cortex. Ketamine induced reductions in SI associated with reductions in rCMRglu in infralimbic cortex and increases in rCMRglu in cluster including lingual gyrus, occipital cortex and cerebellum

Authors, year*	Mental disorder	SA group/ SI group	Groups w/o SA and/or SI	Female n (%)**	Age	Methods	Findings***
MAGNETIC RESONANCE SPECTROSCOPY STUDIES							
Suicide attempt studies							
Jollant et al 2017[87]	past MDD	15 SA	10 DC, 33 HC	35 (60)	15 -55	Proton MRS;. Metabolites: glutamate, glutamine, N-acetylaspartate, myo-inositol, aspartate, glutathione, GABA, N-acetylaspartylglutamate, total choline. ROI: right dorsal PFC	SA vs DC: no significant differences. Choline levels positively correlated with current suicidal ideation
Prescot et al 2018[88]	history or current MDD, PTSD and/or SUD	57 with SA or SI	24 DC	16 (20)	SA+SI: 37.2 (9.1), DC: 36.2 (9.7)	Proton Magnetic Resonance Spectroscopy. Metabolites: GABA, N-acetylaspartylglutamate, glutamine, glutamate, creatine. ROI: dorsal ACC	SA vs DC: no significant differences. In females only, ↓ GABA in SA+SI compared to DC but no longer significant after correcting for age differences
Rocha et al 2015[89]	past BD	19 SA	21 DC, 22 HC	41 (66)	SA: 39.8 (11.4), DC: 42.0 (8.6), HCL 37.7 (13.5)	Proton MRS; Metabolites: N-acetylaspartate, choline, creatine, myo-inositol. ROI: medial OFC	SA vs DC: no significant differences
Suicidal ideation studies							
Gabbay et al 2017[90]	MDD		44 DC, 36 HC	46 (50)	12-21	Proton MRS; Metabolites: GABA and GLX. ROI: rostral ACC	No correlation between GABA and GLX levels in rostral ACC and SI

Symbols & Abbreviations: *Full citations can be found in the reference list below; **Percentages are rounded to the nearest whole number; ***Results are reported for SA or SI in comparison with diagnostic controls. If no diagnostic controls were included in the study, results based on SA or SI compared to healthy controls are reported.
ACC: anterior cingulate cortex; **AD**: adjustment disorder; **ANX**: anxiety disorder; **BD**: bipolar disorder; **BPD**: borderline personality disorder; **DAT**: dopamine transporter; **DC**: diagnostic controls; **DLPFC**: dorsolateral prefrontal cortex; **DPFC**: dorsolateral prefrontal cortex; **FDG**: fludeoxyglucose; **GABA:** gamma-aminobutyric acid; **GLX**: glutamate + glutamine; **HC**: healthy controls; **HL**: high lethality; **IFG**: inferior frontal gyrus; **LL**: low lethality; **MAO-A**: monoamine oxidase A; **MDD**: major depressive disorder; **MRS**: magnetic resonance spectroscopy; **ND:** not detailed; **OFC**: orbitofrontal cortex; **PET**: positron emission tomography; **PD**: panic disorder; **PFC**: prefrontal cortex; **PsD**: psychotic disorder; **rCBF**: regional cerebral blood flow; **rCMRglu:** regional cerebral metabolic rate for glucose; **RLPFC**: rostrolateral prefrontal cortex; **SA**: suicide attempt; **SERT**: serotonin transporter; **SI**: suicidal ideation; **SP**: social phobia; **SPECT**: single photon emission computed tomography; **SUD**: substance use disorder; **TSPO**: translocator protein, **VMPFC**: ventromedial prefrontal cortex; **w/o:** without; **5-HT**: serotonin, **5-HTT**: serotonin transporter

Supplementary Table S3. Findings from Functional Imaging Studies of Suicidal Thoughts and Behaviors

Authors, year*	Mental disorder	SA group/ SI group	Groups w/o SA and/or SI	Female n (%)**	Age	Methods	Findings***
**FUNCTIONAL MAGNETIC RESONANCE IMAGING STUDIES**							
Suicide attempt studies							
Resting State fMRI							
Cao et al 2016[3]	DD	35 SA	18 DC, 47 HC	66 (66)	SA: 20.63 (3.65), DC: 21.39 (3.05), HC: 20.53 (1.84)	WB fractional zALFF	SA vs DC: ↑ zALFF in right superior temporal gyrus, left middle temporal gyrus, left middle occipital gyrus, left angular gyrus, ↓ zALFF in left RLPFC. In SA: negative correlation impulsivity and zALFF in left RLPFC
Cao et al 2015[91]	No DX	19 SA	20 HC	22 (56)	SA: 19.8 (1.6), HC: 20.3 (1.7)	WB ReHo	SA vs HC: ↓ ReHo in left fusiform gyrus, lateral OFC (BA47), hippocampus, right angular gyrus, bilateral parahippocampal gyrus, DLPFC (BA46) and cerebellum, ↑ ReHo in right inferior parietal lobe, left precuneus, right medial OFC (BA11)
Zhang et al 2016[92]	DD	35 SA	18 DC, 47 HC	66 (66)	SA: 20.63 (3.65), DC: 21.26 (3.02), HC: 20.48 (1.86)	ICA of DMN	SA vs DC: ↓ FC in the right precuneus, ↑ FC in the left lingual gyrus and left cerebellum
Kang et al 2017[93]	MDD	19 SA	19 DC	20 (53)	SA: 42.0 (10.8), DC: 41.1 (15.2)	Amygdala seed-based FC	SA vs DC: ↑ FC left amygdala with right insula and left OFC (BA11), ↑ FC right amygdala with left middle temporal gyrus. In SA: positive correlation between SI and right amygdala FC with right parahippocampal gyrus
Cognitive control							
Pan et al 2011[94]	MDD	15 SA	15 DC, 14 HC	25 (57)	12-17	Go-no-go response inhibition, WB	SA vs DC: ↓ dorsal ACC and insula activation during response inhibition, driven by greater activity in DC compared to both SA and HC (i.e., no evidence for abnormal response inhibition circuitry in SA)
Richard-Devantoy et al 2016[95]	MDD	25 SA	22 DC, 27 HC	47 (61)	18-55	Go-no-go response inhibition, WB	SA vs DC: no significant differences. Suicidal intent was positively associated with thalamus activity during response inhibition
Minzenberg et al 2014[96]	SCZ	8 SA+SI, 10 SI only	17 DC	10 (26)	18-50	Continuous performance, ROI frontal cortex	SA+SI vs SI: ↓ goal-related left dorsal premotor cortex (BA6) activity. SI vs no-SI: ↓ goal-related ventral ACC, VMPFC, VLPFC, DLPFC (BA9), RMPFC (BA10), RLPFC (BA10) extending to DMPFC (BA8), dorsal ACC (BA24/32). Intensity of ideation negatively correlated with goal-related activity in DMPFC (BA6/8), dorsal ACC (BA32), DLPFC (BA9), RMPFC (BA10), RLPFC (BA10), IFG (BA44/45)

Authors, year*	Mental disorder	SA group/ SI group	Groups w/o SA and/or SI	Female n (%)**	Age	Methods	Findings***
Minzenberg et al 2015[97]	MD-P	8 SA+SI, 8 SI only	14 DC	13 (43)	18-50	Continuous performance, WB	SA+SI vs SI: ↓ activity in PCC, cuneus and precuneus and ↑ activity right OFC (BA47), right RLPFC (BA10), premotor cortex (BA6), DLPFC (BA9/46), insula during goal-representation. Positive correlation between intensity of SI and goal-related IFG (BA45), lateral and medial OFC (BA11/47), insula and dorsal striatum activity
Minzenberg et al 2015[98]	SCZ	8 SA+SI, 7 SI only	17 DC	6 (19)	18-50	Continuous performance, dorsal ACC seed-based FC	SA+SI vs SI: ↓ dorsal ACC FC with RMPFC and RLPFC (BA10), DMPFC (BA8), DLPFC (BA9), dorsal ACC (BA32), IFG (BA45), superior temporal gyrus, middle temporal gyrus, precuneus, and PCC during conflict monitoring. SI vs no-SI: ↑ dorsal ACC-precuneus FC during conflict monitoring. Intensity of ideation was positively correlated with dorsal ACC FC with paracentral lobe, precuneus, left caudate, right putamen, right lateral globus pallidus and left thalamus
Minzenberg et al 2016[99]	MD-P	8 SA+SI, 8 SI only	14 DC	13 (43)	18-50	Continuous performance, dorsal ACC seed-based FC	SA+SI vs SI: ↑ dorsal ACC FC with left DLPFC (BA9), frontal motor areas (BA4/6), inferior temporal gyrus, middle temporal gyrus, dorsal ACC (BA24/32) during conflict monitoring. SI vs no-SI: ↑ dorsal ACC FC with left DLPFC (BA9), DMPFC (BA8), premotor cortex (BA6), superior parietal cortex, inferior parietal cortex, superior temporal gyrus, middle temporal gyrus. Intensity of ideation positively correlated with dorsal ACC FC with bilateral premotor cortex (BA6), inferior and superior parietal cortex, middle and inferior temporal gyri, middle occipital gyrus and occipital regions, negatively correlated with IFG OFC (BA11/47), insula, putamen, globus pallidum, premotor area (BA6), somatosensory cortex (BA5/7) during conflict monitoring
Minzenberg et al 2015[100]	SCZ	8 SA (all past SI)	9 DC (all past SI)	ND	18-50	Stroop, ROI frontal cortex	SA vs DC: ↑ left DPFC (BA6/8) during cognitive control.
Vanyukov et al 2016[101]	MDD	13 SA	13 DC, 22 HC	30 (63)	46-90	Delay discounting, WB	SA vs DC: ↓ left DLPFC (BA9) activation with increasing value of smaller immediate reward, with a larger decrease in people with better planned SA. Longer versus shorter delay of delayed reward associated with ↓ left parahippocampal gyrus and middle occipital gyrus activation during trials
Decision making and reward processing							
Pan et al 2013[102]	MDD	15 SA	14 DC, 13 HC	23 (55)	12-17	Iowa gambling, WB	SA vs DC: ↓ right thalamus activation during risky choices. No association between activity in thalamus and lethality of attempt and severity of SI

Authors, year*	Mental disorder	SA group/ SI group	Groups w/o SA and/or SI	Female n (%)**	Age	Methods	Findings***
Olié et al 2015[103]	MDD	15 SA	23 DC, 35 HC	0 (0)	18-60	Iowa gambling, ROI OFC, VLPFC, MPFC, ACC, DPFC	SA vs DC: ↓ activation in left DPFC (BA8/9/46) during risky choices, ↑ activation in bilateral DPFC, right OFC (BA11/47), right dorsal and ventral ACC (BA24/32) during winning
Baek et al 2017[104]	past MDD	10 SA	12 DC, 22 HC	24 (55)	18-44	Risk and loss aversion, ROI striatum, OFC, VMPFC, ventral ACC, midbrain	SA vs DC: ↓ subgenual ACC (BA25) activity in response to potential gain. In SA: insula activity correlated negatively with the subjective value of probabilistic gain and loss
Jollant et al 2010[105]	MDD	13 SA	12 DC, 15 HC	0 (0)	22-59	Iowa gambling, ROI OFC, ACC, occipital cortex, precuneus/angular gyrus, thalamus, cerebellum, caudate, cuneus, superior frontal gyrus, parietal cortex	SA vs DC: ↓ lateral OFC (BA47) and occipital cortex activation during risky choices. No differences in activation during gain vs loss trials. No associations with SI
Dombrovski et al 2013[106]	MDD	15 SA	18 DC, 20 HC	31 (59)	60+	Probabilistic reversal learning, WB	SA vs DC: ↓ activity in ventral ACC (BA24/25/32) during expected reward. Poor planning of attempt associated with ↓ activity of paralimbic network (ventral ACC/VMPFC, PCC, precuneus) during expected reward
Memory							
Reisch et al 2010[107]	SR-depression	8 SA	ND	8 (100)	SA: 38.5 (13.1)	Recall of a mental pain, suicide action and neutral conditions using autobiographical scripts of a recent episode of SA, WB	Recall of own suicidal episodes (mental pain and suicide action) vs neutral condition: ↓ activation in left DLPFC (BA46), right RLPFC (BA10), left DMPFC (BA6), ↑ right parahippocampal gyrus, right cuneus, left middle temporal gyrus and cerebellum. Recall of suicide action vs mental pain: ↑ activation in the left DMPFC (BA6), right dorsal ACC (BA32) and left hippocampus
Silvers et al 2016[108]	BPD	46 SA	14 DC	60 (100)	SA: 30.0 (9.8), DC: 26.7 (5.0)	Reappraisal of negative autobiographical memories, ROI lateral OFC	SA vs DC: ↑ lateral OFC activation during both reappraisal and immersion, and ↓ precuneus and cuneus activation during reappraisal of memories. In SA: regulation success associated with greater cuneus and precuneus activity
Emotion processing							
Pan et al 2013[109]	MDD	14 SA	15 DC, 15 HC	25 (57)	12-17	Viewing of angry, happy and neutral faces, WB activity and right dorsal ACC seed-based FC	SA vs DC: ↑ activity in right dorsal ACC, bilateral primary sensory cortex, left DLPFC (BA9), right middle temporal gyrus, ↓ activity in insula and ↓ FC between dorsal ACC and bilateral insula in response to angry faces. In DC: suicidal ideation was negatively correlated with left DLPFC activation during angry faces
Johnston et al 2017[4]	BD	26 SA	42 DC, 45 HC	43 (63)	14-25	Viewing of happy, neutral and fearful faces, amygdala seed-based FC	SA vs DC: ↓ FC amygdala with left OFC (BA11/47), RPFC (BA10), ventral ACC (BA32) in response to happy and neutral faces. SA lethality associated with ↓ FC amygdala with left ventral PFC in response to happy, neutral and fearful faces. In SA: SI was negatively correlated with FC between amygdala and RPFC (BA10)

Authors, year*	Mental disorder	SA group/ SI group	Groups w/o SA and/or SI	Female n (%)**	Age	Methods	Findings***
Olié et al 2015[103]	MDD	15 SA	23 DC, 35 HC	0 (0)	18-60	Viewing of angry, happy, sad and neutral faces, ROI OFC, VLPFC, MPFC, ACC, DPFC	SA vs DC: ↑ activation in left OFC and VLPFC in response to angry faces, and ↓ activation in right ACC in response to sad faces
Kim et al 2017[110]	DD	14 SA	22 HC	26 (72)	20-47	Viewing of angry, happy, sad and neutral faces, and pictures of suicidal means, WB	SA vs HC: ↑ activation in the left DLPFC, IFG, thalamus and PCC when viewing knives versus natural landscapes. No differences in activity while viewing emotional faces
Jollant et al 2008[111]	MDD	13 SA	14 DC, 16 HC	0 (0)	SA: 40.3 (11.3), DC: 43.9 (10.6), HC: 32.4 (9.8)	Viewing of angry, happy and neutral faces, WB	SA vs DC: ↑ activation in in right lateral OFC (BA47) and ↓ activation in right DMPFC (BA6) in response to intense angry faces, ↑ activation in right rostral ACC (BA32) and ↓ activation in right cerebellum to mild happy faces, ↑ activation in right cerebellum in response to mild angry faces
Vanyukov et al 2015[112]	MDD	18 SA	13 DC, 18 HC	26 (53)	60+	Angry and fearful faces versus shape matching, WB	SA vs DC: no differences. In SA, higher activation of IFG (BA44/45) while matching angry faces was associated with poorer planning of attempt
Self-referential processing							
Quevedo et al 2016[113]	DD	43 HS	39 LS, 37 HC	72 (61)	HS: 14.9 (1.6), LS: 14.9 (1.8), HC: 14.5 (1.5)	Emotional Self-Other Morph-Query (ESOM-Q), WB	HS vs LS: ↓ activity in RMPFC (BA10) and in a cluster including parahippocampus, hippocampus, and amygdala during happy self faces versus happy other faces. When controling for depression severity: HS vs LS: ↓ PCC/precuneus and rostral ACC/BA10 during self faces.
Social exclusion							
Olié et al 2017[114]	past MD	36 SA	41 DC, 28 HC	105 (100)	19-54	Social exclusion (Cyberball), WB	SA vs DC: ↓ activation in left supramarginal gyrus and posterior insula during social exclusion
Suicidal ideation studies							
Resting State fMRI							
Cullen et al 2014[115]	MDD	ND	41 DC, 29 HC	54 (77)	MDD: 15.7 (2), HC: 16.0 (2)	Amygdala seed-based FC	No significant correlation between amygdala FC and suicidal ideation
Ordaz et al 2018[116]	MDD	40 SI (33% past SA)		30 (75)	14-17	ICA of DMN, ECN, and SN	↓ Network coherence of left ECN, anterior DMN and SN associated with ↑ lifetime SI, only association with left ECN remained after controlling for depressive and anxiety symptom severity. Left ECN also associated with past SA at a trend-level
Chase et al 2017[117]	MDD	34 SI (18 past SA)	40 HC	50 (68)	18-35	PCC seed-based FC	SI vs HC: ↑ dorsal PCC FC with left middle temporal gyrus. SA vs no-SA: ↑ dorsal PCC FC with left IFG
Du et al 2017[118]	MDD	28 SI	20 DC, 30 HC	55 (71)	SI: 32.5 (9.9), DC: 37.1 (10.6), 35.7 (10.2)	Rostral ACC seed-based FC	SI vs DC: ↓ FC right rostral ACC to medial OFC and right middle temporal gyrus, finding for right middle temporal gyrus remained when corrected for depressive symptom severity

Authors, year*	Mental disorder	SA group/ SI group	Groups w/o SA and/or SI	Female n (%)**	Age	Methods	Findings***
Kim et al 2017[119]	MDD	23 SI (7 past SA)	23 DC, 36 HC	68 (83)	SA: 47, SI: 57, DC: 52.7, HC: 56.5	Graph theory and network-based analysis	SI vs DC: ↓ FC in a network including left lateral OFC, left and right caudate, right putamen, left middle temporal gyrus, left and right thalamus and left postcentral gyrus. Node strength, clustering coefficient, regional efficiency of lateral OFC, left and right thalamus negatively correlated with SI
Cognitive control							
Matthews et al 2012[120]	MDD, PTSD, TBI	13 SI	13 DC	0 (0)	SI: 29.5 (4.7), DC: 27.1 (3.6)	Stop signal response inhibition, WB	SI vs DC: ↑ left dorsal ACC (BA24), RLPFC (BA10), DLPFC (BA9/46), supramarginal gyrus, OFC (BA11), DMPFC (BA6) and superior temporal gyrus during error processing
Lee et al 2015[121]	SCZ	ND	28 DC, 17 HC	8 (18)	SH: 43.6 (11.3), DC: 38.9 (7.3), HC: 37.9 (12.9)	Go-no-go response inhibition, WB	Positive correlation right DLPFC (BA9) activity during response inhibition and current SI in self-harm but no association in no-self-harm group
Zhang et al 2013[122]	SCZ	14 SI	19 DC, 15 HC	24 (50)	18-45	N-back, dynamic causal modelling with PCC and MPFC seeds	High vs Low risk: ↓ no difference in activation of and connectivity between VMPFC and PCC. VMPFC activity was positively related with suicide risk
Decision making and reward processing							
Quevedo et al 2017[123]	MDD	ND	38 DC, 30 HC	44 (65)	DC: 30.7 (7.7), HC: 32.0 (6.1)	Card guessing, ventral striatum seed-based FC	Positive correlation between SI and left ventral striatum FC with DMPFC, DLPFC and dorsal ACC during loss trials
Motor control							
Marchand et al 2012[124]	MDD	5 SA	17 DC	0 (0)	22-45	Motor activation, putamen seed-based FC	Positive correlation between SI and left putamen FC with left DMPFC and right putamen, and right putamen FC with left putamen. Left putamen FC with DMPFC also associated with depressive symptom severity
Marchand et al 2013[125]	MDD, BD	22 SI	18 DC	ND	21-45	Motor activation, PCC seed-based FC	Positive correlation between SI and PCC FC with left DLPFC, DMPFC and IFGs in MDD but not BD. PCC FC with IFG and DLPFC also associated with depressive symptom severity
Marchand et al 2011[126]	BD-II	10 SI	6 DC, 19 HC	0 (0)	BD-II: 32.9 (7.5), HC: 33.7 (12.5)	Motor activation, ROI putamen	Negative correlations between a history of SI and activation in left putamen
Emotion processing							
Marchand et al 2011[127]	BD	10 past SI (3 past SA)	6 DC, 19 HC	0 (0)	21-60	Viewing of happy, fearful and neutral faces, ROI amygdala and subgenual ACC	No correlation between brain activity during the task and history of SI

Authors, year*	Mental disorder	SA group/ SI group	Groups w/o SA and/or SI	Female n (%)**	Age	Methods	Findings***
Just et al 2018[128]	No DX	17 SI (9 past SA)	17 HC	26 (77)	SI: 22.9 (3.6), HC: 22.1 (2.8)	Neurosemantic analyses of concepts related to suicide, positive and negative affect, machine learning on voxels with stable semantic tuning curves	SI could be discriminated from HC with 91% accuracy. Most discriminating regions were the left VMPFC, left DMPFC extending to dorsal ACC, right middle temporal gyrus, left inferior parietal cortex, and left IFG. SI+SA group could be discriminated from SI without SA with 94% accuracy, with most discriminating regions including the left VMPFC, left DMPFC extending to dorsal ACC, right middle temporal gyrus
Emotion regulation							
Miller et al 2018[129]	No DX	14 SI (4 past SA)	32 without SI	29 (63)	13-20	Emotion regulation, WB	SI vs no-SI: ↓ activity in thalamus, IFG/DLPFC (BA44/9), temporoparietal junction and cerebellum and ↑ activity in temporal pole during passive viewing of negative pictures, ↑ activity in DLPFC (BA9) during regulation of negative emotional pictures
NEAR-INFRARED SPECTROSCOPY STUDIES							
Suicide attempt studies							
Tsujii et al 2017[130]	MDD	30 SA	38 DC, 40 HC	69 (64)	SA: 37.6 (10.0), DC: 38.8 (9.7), HC: 38.2 (10.2)	Verbal fluency	SA vs DC: ↓ verbal fluency task induced changes in mean oxy-HB in left precentral gyrus
Suicidal ideation studies							
Pu et al 2015[131]	MDD	31 SI	36 DC, 67 HC	76 (57)	SI: 57.3 (15.7), DC: 58.7 (16.5), HC: 58.1 (17.8)	Verbal fluency	SI vs DC: ↓ verbal Fluency task induced changes in oxy-Hb in right DLPFC, lateral OFC and right RLPFC

Symbols & Abbreviations: *Full citations can be found in the reference list below; **Percentages are rounded to the nearest whole number; ***Results are reported for SA or SI in comparison with diagnostic controls. If no diagnostic controls were included in the study, results based on SA or SI compared to healthy controls are reported.

ACC: anterior cingulate cortex; **BA:** Broadman's Area; **BD**: bipolar disorder; **BD-II**: bipolar II disorder; **BPD**: borderline personality disorder; **DC**: diagnostic controls; **DD**: depressive disorder; **DLPFC**: dorsolateral prefrontal cortex; **DMN**: default mode network; **DMPFC**: dorsomedial prefrontal cortex; **DX**: diagnosis; **ECN**: executive control network; **FC**: functional connectivity; **HC**: healthy controls; **HS**: high suicidality; **ICA**: independent component analysis; **IFG**: inferior frontal gyrus; **LS**: low suicidality; **MDD**: major depressive disorder, **MD-P**: psychotic mood disorder, **MPFC**: medial prefrontal cortex; **ND:** not detailed; **OFC**: orbitofrontal cortex; **oxy-HB:** oxygen-hemoglobin; **PCC**: posterior cingulate cortex; **PFC**: prefrontal cortex; **PTSD**: posttraumatic stress disorder; **ReHo**: regional homogeneity; **RLPFC**: rostrolateral prefrontal cortex; **RMPFC**: rostromedial prefrontal cortex; **SA**: suicide attempt; **SCZ**: schizophrenia; **SI**: suicidal ideation; **SN**: salience network; **SR-depression**: self-reported depression; **ROI**: region of interest; **TBI**: traumatic brain injury; **VLPFC**: ventrolateral prefrontal cortex, **VMPFC**: ventromedial prefrontal cortex; **WB**: whole brain; **w/o:** without; **zALFF**: z score amplitude of low frequency fluctuations

REFERENCES

1 Goodman M, Hazlett EA, Avedon JB, Siever DR, Chu KW, New AS. Anterior cingulate volume reduction in adolescents with borderline personality disorder and co-morbid major depression. *J Psychiatr Res* 2011; **45**: 803–807.

2 Fradkin Y, Khadka S, Bessette KL, Stevens MC. The relationship of impulsivity and cortical thickness in depressed and non-depressed adolescents. *Brain Imaging Behav* 2017; **11**: 1515–1525.

3 Cao J, Chen X, Chen J, Ai M, Gan Y, Wang W *et al.* Resting-state functional MRI of abnormal baseline brain activity in young depressed patients with and without suicidal behavior. *J Affect Disord* 2016; **205**: 252–263.

4 Johnston JAY, Wang F, Liu JJ, Blond BN, Wallace A, Liu JJ *et al.* Multimodal neuroimaging of frontolimbic structure and function associated with suicide attempts in adolescents and young adults with bipolar disorder. *Am J Psychiatry* 2017; **174**: 667–675.

5 Pan LA, Ramos L, Segreti AM, Brent DA, Phillips ML. Right superior temporal gyrus volume in adolescents with a history of suicide attempt. *Br J Psychiatry* 2015; **206**: 339–340.

6 Peng H, Wu K, Li J, Qi H, Guo S, Chi M *et al.* Increased suicide attempts in young depressed patients with abnormal temporal-parietal-limbic gray matter volume. *J Affect Disord* 2014; **165**: 69–73.

7 Gosnell SN, Velasquez KM, Molfese DL, Molfese PJ, Madan A, Fowler JC *et al.* Prefrontal cortex, temporal cortex, and hippocampus volume are affected in suicidal psychiatric patients. *Psychiatry Res - Neuroimaging* 2016; **256**: 50–56.

8 Soloff PH, Pruitt P, Sharma M, Radwan J, White R, Diwadkar VA. Structural brain abnormalities and suicidal behavior in borderline personality disorder. *J Psychiatr Res* 2012; **46**: 516–525.

9 Monkul ES, Hatch JP, Nicoletti MA, Spence S, Brambilla P, Lacerda ALT *et al.* Fronto-limbic brain structures in suicidal and non-suicidal female patients with major depressive disorder. *Mol Psychiatry* 2007; **12**: 360–366.

10 Besteher B, Wagner G, Koch K, Schachtzabel C, Reichenbach JR, Schlösser R *et al.* Pronounced prefronto-temporal cortical thinning in schizophrenia: Neuroanatomical correlate of suicidal behavior? *Schizophr Res* 2016; **176**: 151–157.

11 Giakoumatos CI, Mathew IT. Are Structural Brain Abnormalities Associated With Suicidal Behaviour in Patients With Psiychotic Disorder? *J Psychiatr Res* 2014; **47**: 1389–1395.

12 Rüsch N, Spoletini I, Wilke M, Martinotti G, Bria P, Trequattrini A *et al.* Inferior frontal white matter volume and suicidality in schizophrenia. *Psychiatry Res - Neuroimaging* 2008; **164**: 206–214.

13 Matsuo K, Nielsen N, Nicoletti MA, Hatch JP, Monkul ES, Watanabe Y *et al.* Anterior genu corpus callosum and impulsivity in suicidal patients with bipolar disorder. *Neurosci Lett* 2010; **469**: 75–80.

14 Lijffijt M, Rourke ED, Swann AC, Zunta-Soares GB, Soares JC. Illness-course modulates suicidality-related prefrontal gray matter reduction in women with bipolar disorder. *Acta Psychiatr Scand* 2014; **130**: 374–387.

15 Soloff P, White R, Diwadkar VA. Impulsivity, aggression and brain structure in high and low lethality suicide attempters with borderline personality disorder.

Psychiatry Res - Neuroimaging 2014; **222**: 131–139.

16 Aguilar EJ, García-Martí G, Martí-Bonmatí L, Lull JJ, Moratal D, Escartí MJ *et al.* Left orbitofrontal and superior temporal gyrus structural changes associated to suicidal behavior in patients with schizophrenia. *Prog Neuro-Psychopharmacology Biol Psychiatry* 2008; **32**: 1673–1676.

17 Ding Y, Lawrence N, Olié E, Cyprien F, Le Bars E, Bonafé A *et al.* Prefrontal cortex markers of suicidal vulnerability in mood disorders: A model-based structural neuroimaging study with a translational perspective. *Transl Psychiatry* 2015; **5**: e516.

18 Gifuni AJ, Olié E, Ding Y, Cyprien F, le Bars E, Bonafé A *et al.* Corpus callosum volumes in bipolar disorders and suicidal vulnerability. *Psychiatry Res - Neuroimaging* 2017; **262**: 47–54.

19 Gifuni AJ, Ding Y, Olié E, Lawrence N, Cyprien F, Le Bars E *et al.* Subcortical nuclei volumes in suicidal behavior: nucleus accumbens may modulate the lethality of acts. *Brain Imaging Behav* 2016; **10**: 96–104.

20 Harenski CL, Brook M, Kosson DS, Bustillo JR, Harenski KA, Caldwell MF *et al.* Socio-neuro risk factors for suicidal behavior in criminal offenders with psychotic disorders. *Soc Cogn Affect Neurosci* 2017; **12**: 70–80.

21 Nery-Fernandes F, Rocha M V., Jackowski A, Ladeia G, Guimarães JL, Quarantini LC *et al.* Reduced posterior corpus callosum area in suicidal and non-suicidal patients with bipolar disorder. *J Affect Disord* 2012; **142**: 150–155.

22 Vang FJ, Ryding E, Träskman-Bendz L, van Westen D, Lindström MB. Size of basal ganglia in suicide attempters, and its association with temperament and serotonin transporter density. *Psychiatry Res - Neuroimaging* 2010; **183**: 177–179.

23 Baldaçara L, Nery-Fernandes F, Rocha M, Quarantini LC, Rocha GGL, Guimarães JL *et al.* Is cerebellar volume related to bipolar disorder? *J Affect Disord* 2011; **135**: 305–309.

24 Wagner G, Koch K, Schachtzabel C, Schultz CC, Sauer H, Schlösser RG. Structural brain alterations in patients with major depressive disorder and high risk for suicide: Evidence for a distinct neurobiological entity? *Neuroimage* 2011; **54**: 1607–1614.

25 Wagner G, Schultz CC, Koch K, Schachtzabel C, Sauer H, Schlösser RG. Prefrontal cortical thickness in depressed patients with high-risk for suicidal behavior. *J Psychiatr Res* 2012; **46**: 1449–1455.

26 Duarte DGG, Neves M de CL, Albuquerque MR, Turecki G, Ding Y, de Souza-Duran FL *et al.* Structural brain abnormalities in patients with type I bipolar disorder and suicidal behavior. *Psychiatry Res - Neuroimaging* 2017; **265**: 9–17.

27 Benedetti F, Riccaboni R, Poletti S, Radaelli D, Locatelli C, Lorenzi C *et al.* The serotonin transporter genotype modulates the relationship between early stress and adult suicidality in bipolar disorder. *Bipolar Disord* 2014; **16**: 857–866.

28 Lee YJ, Kim S, Gwak AR, Kim SJ, Kang SG, Na KS *et al.* Decreased regional gray matter volume in suicide attempters compared to suicide non-attempters with major depressive disorders. *Compr Psychiatry* 2016; **67**: 59–65.

29 Spoletini I, Piras F, Fagioli S, Rubino IA, Martinotti G, Siracusano A *et al.* Suicidal attempts and increased right amygdala volume in schizophrenia. *Schizophr Res* 2011; **125**: 30–40.

30 Benedetti F, Radaelli D, Poletti S, Locatelli C, Falini A, Colombo C *et al.* Opposite effects of suicidality and lithium on gray matter volumes in bipolar depression. *J Affect Disord* 2011; **135**: 139–147.

31 Colle R, Chupin M, Cury C, Vandendrie C, Gressier F, Hardy P *et al.* Depressed suicide attempters have smaller hippocampus than depressed patients without suicide attempts. *J Psychiatr Res* 2015; **61**: 13–18.

32 Dombrovski AY, Siegle GJ, Szanto K, Clark L, Reynolds CF, Aizenstein H. The temptation of suicide: Striatal gray matter, discounting of delayed rewards, and suicide attempts in late-life depression. *Psychol Med* 2012; **42**: 1203–1215.

33 Cyprien F, Courtet P, Malafosse A, Maller J, Meslin C, Bonafé A *et al.* Suicidal behavior is associated with reduced corpus callosum area. *Biol Psychiatry* 2011; **70**: 320–326.

34 Hwang JP, Lee TW, Tsai SJ, Chen TJ, Yang CH, Lirng JF *et al.* Cortical and subcortical abnormalities in late-onset depression with history of suicide attempts investigated with MRI and voxel-based morphometry. *J Geriatr Psychiatry Neurol* 2010; **23**: 171–184.

35 Lopez-Larson M, King JB, McGlade E, Bueler E, Stoeckel A, Epstein DJ *et al.* Enlarged thalamic volumes and increased fractional anisotropy in the thalamic radiations in veterans with suicide behaviors. *Front Psychiatry* 2013; **4**: 1–13.

36 Jia Z, Huang X-Q, Wu Q-Z, Zhang T-J, Lui S, Zhang J *et al.* High-field magnetic resonance imaging of suicidality in patients with major depressive disorder. *Am J Psychiatry* 2010; **167**: 1381–1390.

37 Kim B, Oh J, Kim MK, Lee S, Tae WS, Kim CM *et al.* White matter alterations are associated with suicide attempt in patients with panic disorder. *J Affect Disord* 2015; **175**: 139–146.

38 Rentería ME, Schmaal L, Hibar DP, Couvy-Duchesne B, Strike LT, Mills NT *et al.* Subcortical brain structure and suicidal behaviour in major depressive disorder: A meta-analysis from the ENIGMA-MDD working group. *Transl Psychiatry* 2017; **7**: e1116.

39 Thomas LA, de Bellis MD. Pituitary Volumes in Pediatric Maltreatment-Related Posttraumatic Stress Disorder. *Biol Psychiatry* 2004; **55**: 725–758.

40 Taylor WD, Boyd B, McQuoid DR, Kudra K, Saleh A, MacFall JR. Widespread white matter but focal gray matter alterations in depressed individuals with thoughts of death. *Prog Neuro-Psychopharmacology Biol Psychiatry* 2015; **62**: 22–28.

41 Caplan R, Siddarth P, Levitt J, Gurbani S, Shields WD, Sankar R. Suicidality and brain volumes in pediatric epilepsy. *Epilepsy Behav* 2010; **18**: 286–290.

42 Ehrlich S, Noam GG, Lyoo IK, Kwon BJ, Clark MA, Renshaw PF. Subanalysis of the location of white matter hyperintensities and their association with suicidality in children and youth. *Ann N Y Acad Sci* 2003; **1008**: 265–268.

43 Ehrlich S, Noam GG, Lyoo IK, Kwon BJ, Clark MA, Renshaw PF. White matter hyperintensities and their associations with suicidality in psychiatrically hospitalized children and adolescents. *J Am Acad Child Adolesc Psychiatry* 2004; **43**: 770–776.

44 Ehrlich S, Breeze JL, Hesdorffer DC, Noam GG, Hong X, Alban RL *et al.* White matter hyperintensities and their association with suicidality in depressed young adults. *J Affect Disord* 2005; **86**: 281–287.

45 Pompili M, Ehrlich S, De Pisa E, Mann JJ, Innamorati M, Cittadini A *et al.* White matter hyperintensities and their associations with suicidality in patients with major affective disorders. *Eur Arch Psychiatry Clin Neurosci* 2007; **257**: 494–499.

46 Pompili M, Innamorati M, Mann JJ, Oquendo MA, Lester D, Del Casale A *et al.* Periventricular white matter hyperintensities as predictors of suicide attempts in bipolar disorders and unipolar depression. *Prog Neuro-Psychopharmacology Biol Psychiatry* 2008; **32**: 1501–1507.

47 Ahearn EP, Jamison KR, Steffens DC, Cassidy F, Provenzale JM, Lehman A *et al.* MRI correlates of suicide attempt history in unipolar depression. *Biol*

Psychiatry 2001; **50**: 266–270.

48 Sachs-Ericsson N, Hames JL, Joiner TE, Corsentino E, Rushing NC, Palmer E *et al.* Differences between suicide attempters and nonattempters in depressed older patients: Depression severity, white-matter lesions, and cognitive functioning. *Am J Geriatr Psychiatry* 2014; **22**: 75–85.

49 Lischke A, Domin M, Freyberger HJ, Grabe HJ, Mentel R, Bernheim D *et al.* Structural Alterations in the Corpus Callosum Are Associated with Suicidal Behavior in Women with Borderline Personality Disorder. *Front Hum Neurosci* 2017; **11**: 1–10.

50 Lee SJ, Kim B, Oh D, Kim MK, Kim KH, Bang SY *et al.* White matter alterations associated with suicide in patients with schizophrenia or schizophreniform disorder. *Psychiatry Res - Neuroimaging* 2016; **248**: 23–29.

51 Mahon K, Burdick KE, Wu J, Ardekani BA, Szeszko PR. Relationship between suicidality and impulsivity in bipolar I disorder: A diffusion tensor imaging study. *Bipolar Disord* 2012; **14**: 80–89.

52 Olvet DM, Peruzzo D, Thapa-Chhetry B, Sublette ME, Sullivan GM, Oquendo MA *et al.* A diffusion tensor imaging study of suicide attempters. *J Psychiatr Res* 2014; **51**: 60–67.

53 Jia Z, Wang Y, Huang X, Kuang W, Wu Q, Lui S *et al.* Impaired frontothalamic circuitry in suicidal patients with depression revealed by diffusion tensor imaging at 3.0 T. *J Psychiatry Neurosci* 2014; **39**: 170–177.

54 Cyprien F, de Champfleur NM, Deverdun J, Olié E, Le Bars E, Bonafé A *et al.* Corpus callosum integrity is affected by mood disorders and also by the suicide attempt history: A diffusion tensor imaging study. *J Affect Disord* 2016; **206**: 115–124.

55 Bijttebier S, Caeyenberghs K, van den Ameele H, Achten E, Rujescu D, Titeca K *et al.* The Vulnerability to Suicidal Behavior is Associated with Reduced Connectivity Strength. *Front Hum Neurosci* 2015; **9**: 632.

56 Myung W, Han CE, Fava M, Mischoulon D, Papakostas GI, Heo JY *et al.* Reduced frontal-subcortical white matter connectivity in association with suicidal ideation in major depressive disorder. *Transl Psychiatry* 2016; **6**: e835--8.

57 Audenaert K, Van Laere K, Dumont F, Slegers G, Mertens J, Van Heeringen C *et al.* Decreased frontal serotonin 5-HT2areceptor binding index in deliberate self-harm patients. *Eur J Nucl Med* 2001; **28**: 175–182.

58 Audenaert K, Goethals I, Van laere K, Lahorte P, Brans B, Versijpt J *et al.* Spect neuropsychological activation procedure with the verbal fluency test in attempted suicide patients. *Nucl Med Commun* 2002; **23**: 907–916.

59 Van Heeringen C, Audenaert K, Van Laere K, Dumont F, Slegers G, Mertens J *et al.* Prefrontal 5-HT2areceptor binding index, hopelessness and personality characteristics in attempted suicide. *J Affect Disord* 2003; **74**: 149–158.

60 Amen DG, Prunella JR, Fallon JH, Amen B, Hanks C. A Comparative Analysis of Completed Suicide Using High Resolution Brain SPECT Imaging. *J Neuropsychiatry Clin Neurosci* 2009; **21**: 430–439.

61 Willeumier K, Taylor D V., Amen DG. Decreased cerebral blood flow in the limbic and prefrontal cortex using SPECT imaging in a cohort of completed suicides. *Transl Psychiatry* 2011; **1**: e28-8.

62 Fountoulakis K, Lacovides A, Fotiou F, Nimatoudis J, Bascialla F, Ioannidou C *et al.* Neurobiological and psychological correlates of suicidal attempts and thoughts of death in patients with major depression. *Neuropsychobiology* 2004; **49**: 42–52.

63 Henningsson S, Borg J, Lundberg J, Bah J, Lindström M, Ryding E *et al.* Genetic Variation in Brain-Derived Neurotrophic Factor Is Associated with Serotonin Transporter but Not Serotonin-1A Receptor Availability in Men. *Biol Psychiatry* 2009; **66**: 477–485.

64 Bah J, Lindström M, Westberg L, Mannerås L, Ryding E, Henningsson S *et al.* Serotonin transporter gene polymorphisms: Effect on serotonin transporter availability in the brain of suicide attempters. *Psychiatry Res - Neuroimaging* 2008; **162**: 221–229.

65 Lindström MB, Ryding E, Bosson P, Ahnlide JA, Rosén I, Träskman-Bendz L. Impulsivity related to brain serotonin transporter binding capacity in suicide attempters. *Eur Neuropsychopharmacol* 2004; **14**: 295–300.

66 Ryding E, Ahnlide JA, Lindström M, Rosén I, Träskman-Bendz L. Regional brain serotonin and dopamine transporter binding capacity in suicide attempters relate to impulsiveness and mental energy. *Psychiatry Res - Neuroimaging* 2006; **148**: 195–203.

67 Soloff PH, Meltzer CC, Becker C, Greer PJ, Kelly TM, Constantine D. Impulsivity and prefrontal hypometabolism in borderline personality disorder. *Psychiatry Res* 2003; **123**: 153–163.

68 Yeh YW, Ho PS, Chen CY, Kuo SC, Liang CS, Yen CH *et al.* Suicidal ideation modulates the reduction in serotonin transporter availability in male military conscripts with major depression: A 4-[18F]-ADAM PET study. *World J Biol Psychiatry* 2015; **16**: 502–512.

69 Soloff PH, Chiappetta L, Mason NS, Becker C, Price JC. Effects of serotonin-2A receptor binding and gender on personality traits and suicidal behavior in borderline personality disorder. *Psychiatry Res* 2014; **222**: 140–148.

70 Soloff PH, Price JC, Meltzer CC, Fabio A, Frank GK, Kaye WH. 5HT2A Receptor Binding is Increased in Borderline Personality Disorder. *Biol Psychiatry* 2007; **62**: 580–587.

71 Cannon DM, Ichise M, Fromm SJ, Nugent AC, Rollis D, Gandhi SK *et al.* Serotonin Transporter Binding in Bipolar Disorder Assessed using [11C]DASB and Positron Emission Tomography. *Biol Psychiatry* 2006; **60**: 207–217.

72 Oquendo MA, Placidi GPA, Malone KM, Campbell C, Keilp J, Brodsky B *et al.* Positron emission tomography of regional brain metabolic responses to a serotonergic challenge and lethality of suicide attempts in major depression. *Arch Gen Psychiatry* 2003; **60**: 14–22.

73 Sullivan GM, Oquendo MA, Milak M, Miller JM, Burke A, Ogden RT *et al.* Positron emission tomography quantification of serotonin1A receptor binding in suicide attempters with major depressive disorder. *JAMA Psychiatry* 2015; **72**: 169–178.

74 Miller JM, Everett BA, Oquendo MA, Ogden RT, Mann JJ, Parsey R V. Positron Emission Tomography Quantification of Serotonin Transporter Binding in Medication-Free Bipolar Disorder. *Synapse* 2016; **70**: 24–32.

75 Yeh YW, Ho PS, Chen CY, Kuo SC, Liang CS, Ma KH *et al.* Incongruent reduction of serotonin transporter associated with suicide attempts in patients with major depressive disorder: A positron emission tomography study with 4-[18F]-ADAM. *Int J Neuropsychopharmacol* 2015; **18**: 1–9.

76 Leyton M, Paquette V, Gravel P, Rosa-Neto P, Weston F, Diksic M *et al.* α-[11C]methyl-L-tryptophan trapping in the orbital and ventral medial prefrontal cortex of suicide attempters. *Eur Neuropsychopharmacol* 2006; **16**: 220–223.

77 Parsey R V., Oquendo MA, Ogden RT, Olvet DM, Simpson N, Huang YY *et al.* Altered serotonin 1A binding in major depression: A [carbonyl-C-11] WAY100635 positron emission tomography study. *Biol Psychiatry* 2006; **59**: 106–113.

78 Miller JM, Hesselgrave N, Ogden RT, Sullivan GM, Oquendo MA, Mann JJ *et al.* Positron Emission Tomography Quantification of Serotonin Transporter in

Suicide Attempters with Major Depressive Disorder. *Biol Psychiatry* 2013; **74**: 287–295.

79 Nye JA, Purselle D, Plisson C, Voll RJ, Stehouwer JS, Votaw JR *et al.* Decreased brainstem and putamen sert binding potential in depressed suicide attempters using [11C]-zient pet imaging. *Depress Anxiety* 2013; **30**: 902–907.

80 Mann JJ, Metts A V., Ogden RT, Mathis CA, Rubin-Falcone H, Gong Z *et al.* Quantification of 5-HT1Aand 5-HT2Areceptor Binding in Depressed Suicide Attempters and Non-Attempters. *Arch Suicide Res* 2018; **1118**: 1–12.

81 Oquendo MA, Galfalvy H, Sullivan GM, Miller JM, Milak MM, Elizabeth Sublette M *et al.* Positron emission tomographic imaging of the serotonergic system and prediction of risk and lethality of future suicidal behavior. *JAMA Psychiatry* 2016; **73**: 1048–1055.

82 Sublette ME, Milak MS, Galfalvy HC, Oquendo MA, Malone KM, Mann JJ. Regional Brain Glucose Uptake Distinguishes Suicide Attempters from Non-Attempters in Major Depression. *Arch Suicide Res* 2013; **17**: 434–447.

83 Holmes SE, Hinz R, Conen S, Gregory CJ, Matthews JC, Anton-Rodriguez JM *et al.* Elevated Translocator Protein in Anterior Cingulate in Major Depression and a Role for Inflammation in Suicidal Thinking: A Positron Emission Tomography Study. *Biol Psychiatry* 2018; **83**: 61–69.

84 Kolla NJ, Chiuccariello L, Wilson AA, Houle S, Links P, Bagby RM *et al.* Elevated Monoamine Oxidase-A Distribution Volume in Borderline Personality Disorder Is Associated with Severity Across Mood Symptoms, Suicidality, and Cognition. *Biol Psychiatry* 2016; **79**: 117–126.

85 van Heeringen K, Wu GR, Vervaet M, Vanderhasselt MA, Baeken C. Decreased resting state metabolic activity in frontopolar and parietal brain regions is associated with suicide plans in depressed individuals. *J Psychiatr Res* 2017; **84**: 243–248.

86 Ballard ED, Lally N, Nugent AC, Furey ML, Luckenbaugh DA, Zarate CA. Neural correlates of suicidal ideation and its reduction in depression. *Int J Neuropsychopharmacol* 2015; **18**: 1–6.

87 Jollant F, Near J, Turecki G, Richard-Devantoy S. Spectroscopy markers of suicidal risk and mental pain in depressed patients. *Prog Neuro-Psychopharmacology Biol Psychiatry* 2017; **73**: 64–71.

88 Prescot A, Sheth C, Legarreta M, Renshaw PF, McGlade E, Yurgelun-Todd D. Altered Cortical Gamma-Amino Butyric Acid in Female Veterans With Suicidal Behavior: Sex Differences and Clinical Correlates. *Chronic Stress* 2018; **2**. doi:10.1177/2470547018768771.

89 Rocha MV, Nery-Fernandes F, Guimarães JL, De Castro Quarantini L, De Oliveira IR, Ladeia-Rocha GG *et al.* Normal metabolic levels in prefrontal cortex in euthymic bipolar I patients with and without suicide attempts. *Neural Plast* 2015: 165180.

90 Gabbay V, Bradley KA, Mao X, Ostrover R, Kang G, Shungu DC. Anterior cingulate cortex γ-aminobutyric acid deficits in youth with depression. *Transl Psychiatry* 2017; **7**: e1216.

91 Cao J, mei Chen J, Kuang L, Ai M, dong Fang W, Gan Y *et al.* Abnormal regional homogeneity in young adult suicide attempters with no diagnosable psychiatric disorder: A resting state functional magnetic imaging study. *Psychiatry Res - Neuroimaging* 2015; **231**: 95–102.

92 Zhang S, Chen J mei, Kuang L, Cao J, Zhang H, Ai M *et al.* Association between abnormal default mode network activity and suicidality in depressed adolescents. *BMC Psychiatry* 2016; **16**: 1–10.

93 Kang SG, Na KS, Choi JW, Kim JH, Son YD, Lee YJ. Resting-state functional connectivity of the amygdala in suicide attempters with major depressive disorder. *Prog Neuro-Psychopharmacology Biol Psychiatry* 2017; **77**: 222–227.

94 Pan LA, Batezati-Alves SC, Almeida JRC, Segreti A, Akkal D, Hassel S *et al.* Dissociable patterns of neural activity during response inhibition in depressed adolescents with and without suicidal behavior. *J Am Acad Child Adolesc Psychiatry* 2011; **50**: 602–611.

95 Richard-Devantoy S, Olié E, Guillaume S, Courtet P. Decision-making in unipolar or bipolar suicide attempters. *J Affect Disord* 2016; **190**: 128–136.

96 Minzenberg MJ, Lesh TA, Niendam TA, Yoon JH, Rhoades RN, Carter CS. Frontal cortex control dysfunction related to long-term suicide risk in recent-onset schizophrenia. *Schizophr Res* 2014; **157**: 19–25.

97 Minzenberg MJ, Lesh TA, Niendam TA, Yoon JH, Cheng Y, Rhoades RN *et al.* Control-related frontal-striatal function is associated with past suicidal ideation and behavior in patients with recent-onset psychotic major mood disorders. *J Affect Disord* 2015; **188**: 202–209.

98 Minzenberg M, Lesh T, Niendam T, Yoon J, Cheng Y, Rhoades R *et al.* Conflict-related anterior cingulate functional connectivity is associated with past suicidal ideation and behavior in recent-onset schizophrenia. *J Psychiatr Res* 2015; **65**: 95–101.

99 Minzenberg MJ, Lesh T, Niendam T, Yoon JH, Cheng Y, Rhoades R *et al.* Conflict-related anterior cingulate functional connectivity is associated with past suicidal ideation and behavior in recent-onset Psychotic Major Mood Disorders. *J Neuropsychiatry Clin Neurosci* 2016; **28**: 95–101.

100 Minzenberg MJ, Lesh T, Niendam T, Yoon JH, Cheng Y, Rhoades RN *et al.* Frontal motor cortex activity during reactive control is associated with past suicidal behavior in recent-onset schizophrenia. *Crisis* 2015; **36**: 363–370.

101 Vanyukov PM, Szanto K, Hallquist MN, Siegle GJ, Reynolds CF, Forman SD *et al.* Paralimbic and lateral prefrontal encoding of reward value during intertemporal choice in attempted suicide. *Psychol Med* 2016; **46**: 381–391.

102 Pan L, Segreti A, Almeida J, Jollant F, Lawrence N, Brenta D *et al.* Preserved hippocampal function during learning in the context of risk in adolescent suicide attempt. *Psychiatry Res Neuroimaging* 2013; **211**: 112–118.

103 Olié E, Ding Y, Le Bars E, de Champfleur NM, Mura T, Bonafé A *et al.* Processing of decision-making and social threat in patients with history of suicidal attempt: A neuroimaging replication study. *Psychiatry Res - Neuroimaging* 2015; **234**: 369–377.

104 Baek K, Kwon J, Chae JH, Chung YA, Kralik JD, Min JA *et al.* Heightened aversion to risk and loss in depressed patients with a suicide attempt history. *Sci Rep* 2017; **7**: 11228.

105 Jollant F, Lawrence NS, Olie E, O'Daly O, Malafosse A, Courtet P *et al.* Decreased activation of lateral orbitofrontal cortex during risky choices under uncertainty is associated with disadvantageous decision-making and suicidal behavior. *Neuroimage* 2010; **51**: 1275–1281.

106 Dombrovski AY, Szanto K, Clark L, Reynolds CF, Siegle GJ. Reward signals, attempted suicide, and impulsivity in late-life depression. *JAMA Psychiatry* 2013; **70**: 1020–1030.

107 Reisch T, Seifritz E, Esposito F, Wiest R, Valach L, Michel K. An fMRI study on mental pain and suicidal behavior. *J Affect Disord* 2010; **126**: 321–325.

108 Silvers JA, Hubbard AD, Chaudhury S, Biggs E, Shu J, Grunebaum MF *et al.* Suicide attempters with Borderline Personality Disorder show differential orbitofrontal and parietal recruitment when reflecting on aversive memories. *J Psychiatr Res* 2016; **81**: 71–78.

109 Pan LA, Hassel S, Segreti AM, Nau SA, Brent DA, Phillips ML. Differential patterns of activity and functional connectivity in emotion processing neural circuitry to angry and happy faces in adolescents with and without suicide attempt. *Psychol Med* 2013; **43**: 2129–2142.

110 Kim YJ, Park HJ, Jahng GH, Lee SM, Kang WS, Kim SK *et al.* A pilot study of differential brain activation to suicidal means and DNA methylation of

CACNA1C gene in suicidal attempt patients. *Psychiatry Res* 2017; **255**: 42–48.

111 Jollant F, Lawrence NS, Giampietro V, Brammer MJ, Fullana MA, Drapier D *et al.* Orbitofrontal cortex response to angry faces in men with histories of suicide attempts. *Am J Psychiatry* 2008; **165**: 740–748.

112 Vanyukov PM, Szanto K, Siegle GJ, Hallquist MN, Reynolds CF, Aizenstein HJ *et al.* Impulsive traits and unplanned suicide attempts predict exaggerated prefrontal response to angry faces in the elderly. *Am J Geriatr Psychiatry* 2015; **23**: 829–839.

113 Quevedo K, Ng R, Scott H, Martin J, Smyda G, Keener M *et al.* The Neurobiology of Self-Face Recognition in Depressed Adolescents with Low or High Suicidality. *J Abnorm Psychol* 2016; **125**: 1185–1200.

114 Olié E, Jollant F, Deverdun J, De Champfleur NM, Cyprien F, Le Bars E *et al.* The experience of social exclusion in women with a history of suicidal acts: neuroimaging study. *Sci Rep* 2017; **7**: 1–8.

115 Cullen KR, Westlund MK, Klimes-Dougan B, Mueller BA, Houri A, Eberly LE *et al.* Abnormal amygdala resting-state functional connectivity in adolescent depression. *JAMA Psychiatry* 2014; **71**: 1138–1147.

116 Ordaz SJ, Goyer MS, Ho TC, Singh MK, Gotlib IH. Network basis of suicidal ideation in depressed adolescents. *J Affect Disord* 2018; **226**: 92–99.

117 Chase HW, Segreti AM, Keller TA, Cherkassky VL, Just MA, Pan LA *et al.* Alterations of functional connectivity and intrinsic activity within the cingulate cortex of suicidal ideators. *J Affect Disord* 2017; **212**: 78–85.

118 Du L, Zeng J, Liu H, Tang D, Meng H, Li Y *et al.* Fronto-limbic disconnection in depressed patients with suicidal ideation: A resting-state functional connectivity study. *J Affect Disord* 2017; **215**: 213–217.

119 Kim K, Kim SW, Myung W, Han CE, Fava M, Mischoulon D *et al.* Reduced orbitofrontal-thalamic functional connectivity related to suicidal ideation in patients with major depressive disorder. *Sci Rep* 2017; **7**: 15772.

120 Matthews S, Spadoni A, Knox K, Strigo I, Simmons A. Combat-exposed war veterans at risk for suicide show hyperactivation of prefrontal cortex and anterior cingulate during error processing. *Psychosom Med* 2012; **74**: 471–475.

121 Lee KH, Pluck G, Lekka N, Horton A, Wilkinson ID, Woodruff PWR. Self-harm in schizophrenia is associated with dorsolateral prefrontal and posterior cingulate activity. *Prog Neuro-Psychopharmacology Biol Psychiatry* 2015; **61**: 18–23.

122 Zhang H, Wei X, Tao H, Mwansisya TE, Pu W, He Z *et al.* Opposite Effective Connectivity in the Posterior Cingulate and Medial Prefrontal Cortex between First-Episode Schizophrenic Patients with Suicide Risk and Healthy Controls. *PLoS One* 2013; **8**: 1–8.

123 Quevedo K, Ng R, Scott H, Kodavaganti S, Smyda G, Diwadkar V *et al.* Ventral Striatum Functional Connectivity during Rewards and Losses and Symptomatology in Depressed Patients. *Biol Psychol* 2017; **123**: 62–73.

124 Marchand WR, Lee JN, Johnson S, Thatcher J, Gale P, Wood N *et al.* Striatal and cortical midline circuits in major depression: Implications for suicide and symptom expression. *Prog Neuro-Psychopharmacology Biol Psychiatry* 2012; **36**: 290–299.

125 Marchand WR, Lee JN, Johnson S, Gale P, Thatcher J. Differences in functional connectivity in major depression versus bipolar II depression. *J Affect Disord* 2013; **150**: 527–532.

126 Marchand WR, Lee JN, Garn C, Thatcher J, Gale P, Kreitschitz S *et al.* Striatal and cortical midline activation and connectivity associated with suicidal

ideation and depression in bipolar II disorder. *J Affect Disord* 2011; **133**: 638–645.

127 Marchand WR, Lee JN, Garn C, Thatcher J, Gale P, Kreitschitz S *et al.* Aberrant emotional processing in posterior cortical midline structures in bipolar II depression. *Prog Neuro-Psychopharmacology Biol Psychiatry* 2011; **35**: 1729–1737.

128 Just MA, Pan L, Cherkassky VL, Mcmakin D, Cha C, Nock MK *et al.* Machine learning of neural representations of suicide and emotion concepts identifies suicidal youth. *Nat Hum Behav* 2017; **1**: 911–919.

129 Miller AB, McLaughlin KA, Busso DS, Brueck S, Peverill M, Sheridan MA. Neural Correlates of Emotion Regulation and Adolescent Suicidal Ideation. *Biol Psychiatry Cogn Neurosci Neuroimaging* 2018; **3**: 125–132.

130 Tsujii N, Mikawa W, Tsujimoto E, Adachi T, Niwa A, Ono H *et al.* Reduced left precentral regional responses in patients with major depressive disorder and history of suicide attempts. *PLoS One* 2017; **12**: e0175249.

131 Pu S, Nakagome K, Yamada T, Yokoyama K, Matsumura H, Yamada S *et al.* Suicidal ideation is associated with reduced prefrontal activation during a verbal fluency task in patients with major depressive disorder. *J Affect Disord* 2015; **181**: 9–17.
